# Design and analysis of a GaN-based 2D photonic crystal biosensor integrated with machine learning techniques for detection of skin diseases

**DOI:** 10.1038/s41598-025-25893-6

**Published:** 2025-11-25

**Authors:** Harikrishnan N, Sangeetha A

**Affiliations:** Department of Communication Engineering, School of Electronics Engineering, Vellore Institute of Technology, Vellore, Tamil Nadu India

**Keywords:** Biosensors, Vitiligo, Cutis laxa, Photonic crystal, FDTD, Refractive index, Machine learning, Sensitivity, Engineering, Mathematics and computing, Optics and photonics

## Abstract

Photonic crystals are prevalent in the detection of assorted diseases and malignancies such as vitiligo and cutis laxa. A 2D photonic crystal utilizing GaN is demonstrated to detect skin diseases, highlighting its substantial relevance to the photonic sensing community. Various parameters analysed are quality factor, wavelength sensitivity, FWHM, figure of merit and detection limit. The analysis of sensor characteristics demonstrated that GaN is a highly suitable material for detecting vitiligo and cutis laxa. Topology of design is crucial to focus the light on to the sensing region. Opti FDTD tool was used for the design and simulation of the sensor. The photonic band gap was simulated and it was observed that it contained one band gap region. The design provided high transmission efficiency and sensitivity. The various skin abnormalities related to vitiligo and cutis laxa could be easily detected from the results. Machine learning models such as K-nearest neighbor, Random Forest, Support Vector Machine and Multi-Layer Perceptron were adopted to enhance the sensor system to classify the data with higher accuracy.

## Introduction

Optical sensors have wide range of applications in the biomedical field. Photonic crystal (PC) is one of the key component used in detection and early diagnosis of diseases. PCs were used for detection of viruses in blood, diagnosis of cholesterol related diseases, brain tumour detection and cancer cell detection^1–5^. Hyperglycemia diagnosis, detection of bacteria in water, detection of anaemia and detection of SARS-CoV-2 utilizing PCs reported in recent years indicates the potential of PC based structures^6–9^. In 2025, PC was used to implement a multiplexer, and in another work, PC demonstrated high precision in cancer studies^10,11^. Another article reported in 2025, based on refractive index sensing methods, claimed to achieve high sensitivity in tumor detection in the near infrared region^12^. In 2020, ML was utilized in PC based gas sensor to identify greenhouse gases^13^. In 2023, ML was applied to electrochemical biosensor to predict concentration of analytes^14^. Logic gate was implemented using 2D PC with support of FDTD and Artificial neural network (ANN) in 2025^15^. ANN was also used to evaluate dispersion characteristics of PC structure^16^. In another recent work, 2D PC was proposed in the design of perceptron which can enhance computing^17^. Ability of ML and deep learning techniques to reveal information, accelerates analysis of optical data^18^. In an article published in 2025, broader target wavelength range (1725–1765 nm) was selected for PC based sensor with sensitivity in the acceptable range of 219 nm/RIU^19^.

Recent advancements in photonic crystal sensor design have predominantly focused on mid-infrared refractive index sensing^20,21^, spectroscopic gas sensing^22,23^, and optical property optimization using silicon, silicon nitride, or hybrid polymer-based platforms^24,25^. These studies employed slotted PC waveguides^26–29^, point defect PC nanocavities^30,31^, and geometries to enhance Q-factor^32,33^, light confinement, and dispersion control^26^. The applications range from chemical detection to nonlinear optics and liquid sensing^34^. In contrast, the present work introduces a GaN (gallium nitride) based two-dimensional (2D) PC biosensor tailored for biomedical diagnostics, specifically targeting skin disease classification. GaN offers superior thermal stability, wide bandgap, and biocompatibility compared to conventional materials, making it well-suited for clinical environments. Moreover, the integration of machine learning (ML) algorithms enables data-driven classification of biosensor responses, surpassing traditional threshold-based detection methods. This hybrid approach combines nanophotonic precision with computational intelligence, allowing for real-time, and high-sensitivity tissue differentiation. Unlike earlier designs that emphasized passive optical tuning, the proposed sensor leverages active learning models to interpret spectral features, marking a shift toward intelligent, application-specific biosensing platforms.

One of the research topics in the healthcare is the detection of vitiligo. The main characteristic of vitiligo consists of patches of depigmented skin. One of the reasons for loss of pigments is the loss of melanocytes from the skin. Although there are other possible reasons such as environmental factors and sometimes the pigment loss is genetic in nature^35^.

The loss of skin pigments such as melanin, keratin, collagen, epidermis and dermis results in vitiligo. Elastin is another component in the skin which may also cause the loss of melanin^36^. Hence, detection of elastin is also crucial for understanding the loss of pigments from skin. Cutis laxa, a condition which causes loosening of skin may be caused by the decrease in elastin^37^. People get affected by cutis laxa when production of elastin is dysfunctional^38^. In the case of vitiligo, the loss of pigments can cause a patchy appearance to people’s skin. It can be more pronounced in case of dark skinned people. Vitiligo affected patients has more chance to get sunburns. There are many hypotheses for the formation of vitiligo. The reasons of vitiligo can include the effects in the immune system, or it may be due to neuro chemical reactions in the skin. The imbalance in the production of melanocyte cells may also result in vitiligo. According to metabolic hypothesis, oxidative stress can also cause loss of pigments^39^. One of the vitiligo subsets is common vitiligo which is formation of a discolored area of skin in many body parts. The second type is the mixed vitiligo which occurs in paediatrics. The third type of vitiligo subset can be acrofacial vitiligo which mainly affects hands, feet and face. Also, there are some other unclassified categories of vitiligo^40^. The vitiligo types such as generalized, acrofacial and mucosal comes under the category of non-segmental vitiligo. The patients affected with vitiligo may become more anxious about the spreading of the patches and it may also affect their emotional well-being in the society. Also, vitiligo is not gender related because it affects both men and women equally^41^.

The incorporation of artificial intelligence to understand the likelihood of vitiligo would help in the predictive diagnosis of the disease. By deploying state-of-the-art machine learning strategies, vitiligo could be classified as segmental or non-segmental^42^. The deep learning models and methods could also assist dermatologists to assess the condition of vitiligo and provide a timely detection with accuracy^43^. Machine learning tools could become a core component in classifying and detecting diseases such as vitiligo. The strength and augmentation of machine learning techniques lies in their ability provide quick and accurate results^44^. The machine learning algorithm has the ability to understand the patterns such as the wavelength variations or the relative variation of the refractive index with respect to the analytes under observation^45^. In 2022, Support vector machine (SVM) was utilized in PC based sensing approach to support detection of tumor^46^. The machine learning models can be trained for the parameters such as the peak intensity of light and the corresponding wavelength^47^. The machine learning algorithms can be used for the post analysis of the photonic crystal biosensor data. Performance evaluation is the final step which reveals how the model has helped in the accurate classification of the disease^48^. Optimization of the parameters is one of the significant factor in the design of the sensor^49^. The dimensional parameters of the sensor such as wafer dimension, lattice spacing and radius of the crystal can be optimized to attain higher sensitivity and distinct shifts in the wavelengths. The machine learning techniques could be used for identifying the decisive factors and unrevealed relations between the sensor parameters.

Variation in refractive index of material is the parameter that makes difference in the material type and it forms basis for categorizing PCs into different types^50^. One dimensional PC has been employed for diverse biosensing applications such as detection of vitiligo and other skin diseases. Design and fabrication of one dimensional PC lies in arranging materials one over the other. In the case of two dimensional PCs, refractive index profile is different from that of one dimensional PC. We can design 2D PCs by using dielectric rods in the air or by creating air holes in dielectric materials. Three dimensional PCs may also be deployed for detection of vitiligo, but the fabrication process could be challenging compared to two dimensional PC. Firstly, this research paper expresses the design and analysis of the photonic crystal biosensor and the second part of the paper is the application of machine learning for the classification of the data obtained from the sensor. The data to be applied to the machine learning model is obtained by simulation using FDTD tool.

## Design of the proposed PC biosensor for detection of skin diseases

**Fig. 1:**
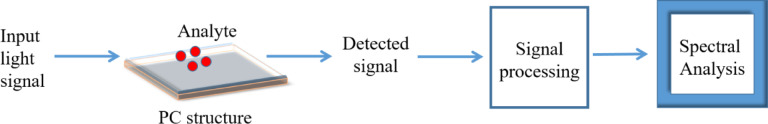
Block diagram of the PC biosensor system.

Design flexibility, higher sensitivity, and seamless integration with on chip formats are among the primary reasons for selecting 2D PCs^51^. The Fig. 1 depicts the representation of the proposed PC biosensor for the detection of skin analytes. The blocks consist of the input light signal, the sensing material, the output signal to be processed and finally the analysis.

Many different sensing materials are under research for performing the bio sensing. Although silicon is one of the commonly used materials as the background material for the sensor, other semiconductor materials may also be used. In this work we have selected GaN (Gallium nitride) as the background material. The sensor design mainly involves the selection of the material. GaN is chosen as the material due to its compatibility with optoelectronic devices. GaN has a lower refractive index value compared to silicon^52^. Selecting the cell size (radius) of the photonic crystal is another important factor in the sensor design. GaN has a higher refractive index value compared to the analytes under investigation. Well defined photonic band gap causes sharp changes in the optical properties upon interaction with an analyte. Stronger light confinement, sharper resonance peaks and enhanced sensitivity in detecting changes in the surrounding environment are some of the advantages of using GaN. PC structures may be designed which can filter out, reflect a particular range of optical wavelengths, or restrict light at distinct optical wavelengths by tailoring the regularity of the crystal, materials opted, sensing region topology, radius of crystal, and refractive index. The sensing area is crucial in the design as it highly affects the output spectrum and the ability of the sensor to detect a particular analyte. Wavelength selection is another important factor in the design of an optical biosensor. Although various wavelengths has been investigated by the researchers such as 1.2 μm,1.5 μm and 0.93 μm. The wavelength at 1.55 μm can be selected as it offers low attenuation.

### FDTD tool

Finite difference time domain (FDTD) approach is widely utilized in PC design to study the optical characteristics^53,54^. The Opti FDTD tool is used to design and perform analysis of the proposed biosensor. It is one of the user friendly tool in which we can specify the sensor parameters such as the dimensions and lattice spacing. The FDTD software is built upon the fundamental Maxwell equations.

The distinct components of propagation of electromagnetic waves are explained using Maxwell’s equations^55^.

1 $$\frac{{\partial E_y}}{{\partial t}}={\text{ }}\frac{{\text{1}}}{{\varepsilon_{0}\varepsilon _r}}\left( {\frac{{\partial H_x}}{{\partial z}} - \frac{{\partial H_z}}{{\partial x}}} \right)$$

2 $$\frac{{\partial H_x}}{{\partial t}}={\text{ }}\frac{{\text{1}}}{{\mu _0\mu _r}}\left( {\frac{{\partial E_y}}{{\partial z}}} \right)$$

3 $$\frac{{\partial H_z}}{{\partial t}}={\text{ }} - \frac{{\text{1}}}{{\mu _0\mu _r}}\left( {\frac{{\partial E_y}}{{\partial x}}} \right)$$

H_x_, E_y_ and H_z_ are constituents of the wave which propagate in z direction. Perpendicular field variations are in the x direction. ε = ε_0_ ε_r_ stands for dielectric permittivity. µ_0_ accounts for magnetic permeability of vacuum. Also, ε_r_ represents permittivity and µ_r_ is the permeability of the material.

**Fig. 2:**
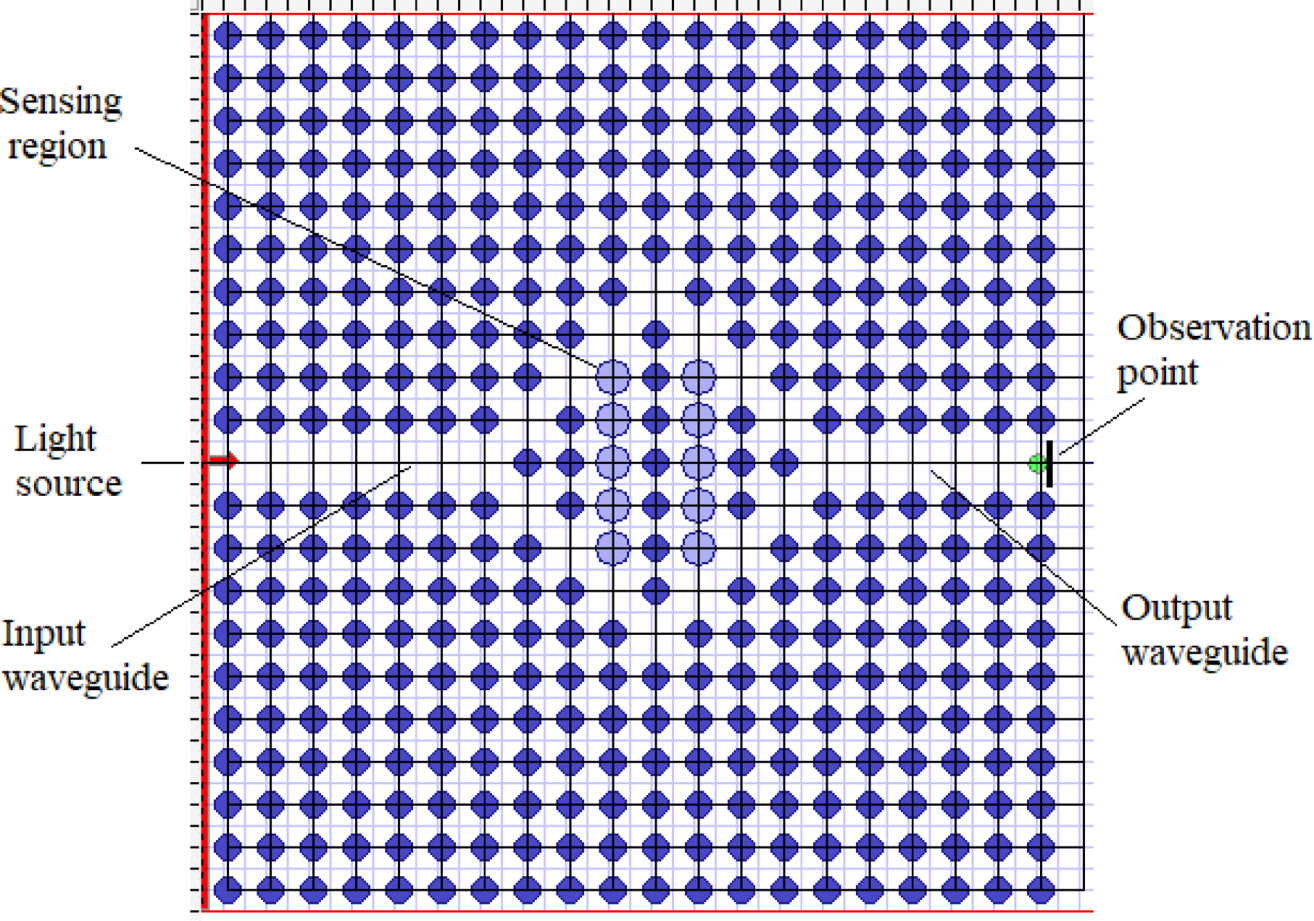
Proposed photonic crystal sensor design.

The sensor design parameters such as wafer dimensions, radius of the crystal, input optical wavelength and base material refractive index are selected in FDTD tool. The wafer dimensions are 21 μm by 21 μm. The PC radius chosen is 0.3 μm, can be identified as dark blue color in Fig. 2. Sensing cell radius is 0.4 μm given in light blue color. The value of index of refraction for GaN falls in the range ~ 2.3–2.4^56^. Real ansd imaginary refractive indices values can be considered for the design. Lattice constant for the design is 1 μm and mesh used is 0.1 μm (delta X) by 0.1 μm (delta Z). TE mode can be selected with time step in the range of 10,000 –15,000. Gaussian modulated continous wave can be selected. The simulation framework can be configured with perfectly matched layers (PML) applied on all boundaries. A uniform grid resolution of 21 μm in both x and y directions is preferred, with the time step governed by a courant factor of 0.5 to ensure numerical stability. Field monitors can be positioned 9 μm away from the defect region. Mesh refinement can be applied within the GaN domains. Convergence is assessed by observing the decay of electromagnetic fields. Each simulation run can be approximately 1–3 min. Parametric sweep can be used to optimize lattice spacing and rod radius.

In Fig. 2, the dark blue colored cells resemble the GaN photonic crystals rods and the light blue coloured cells are the region for analyte under investigation. For different analytes, their index of refraction is different. As a result, the output signal for each analyte has a different value in wavelength. The analyte is placed in the sensor area in the centre position of the sensor. The detection point is kept at the end of the sensor structure. The wavelength of the input light signal is selected as 1550 nm.

GaN is a direct band gap semiconductor which can provide efficient light transmission^57^. Although GaN is used for making light emitting diodes and lasers, it can also be used in photonic crystal biosensing as it has appropriate properties which makes them attractive for the purpose^52^. GaN is selected as it is easier to integrate with other optoelectronic devices.

**Fig. 3:**
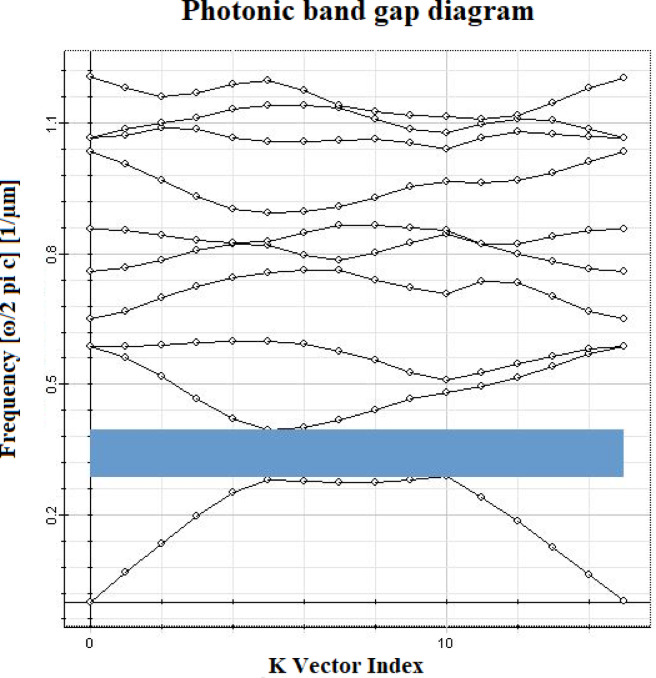
Photonic bandgap diagram of the proposed sensor.

### Photonic band gap

Photonic band gap represents the characteristic of a material which allows photons to be passed over a specific range of wavelength. The Fig. 3 shows photonic band gap diagram of GaN based sensor. For sensor with GaN, photonic band gap value is observed in the range from 0.28 to 0.38 which is shown in light blue color. Photonic band gap range is normalized using the ratio a/λ, where ‘a‘ is lattice constant. Some photons are reflected over a specific wavelength range. This is the reason for development of forbidden band gap.

Design of biosensors using photonic crystals is possible mainly because they have very small dimensions. This compact structure of the photonic crystals makes it suitable candidate to be integrated with optoelectronic devices. The reason behind why the photonic crystal is able to trap the light signal in the small spaces is its photonic band gap. The wavelength of operation is very important in its design, because the output intensity of the crystal varies according to the selected wavelength. The photonic band gap facilitates a significant part to confine light in the biosensor. Photonic band gap of the sensor must be such that it allows the operating wavelength of light to be passed through it^58^. The photonic band gap change depending on the selection of material, material refractive index, cell radius, and lattice spacing.

**Fig. 4:**
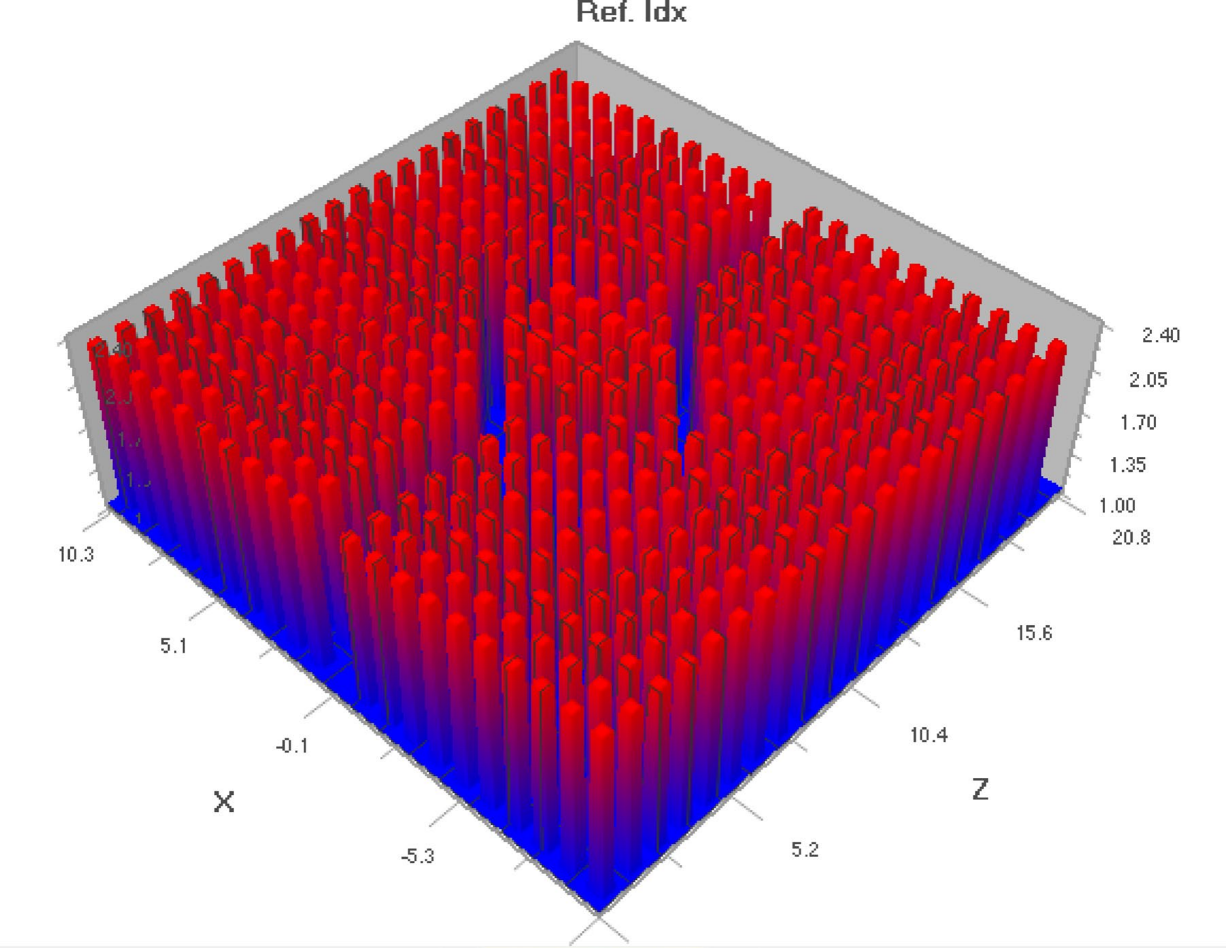
Refractive index profile.

**Fig. 5:**
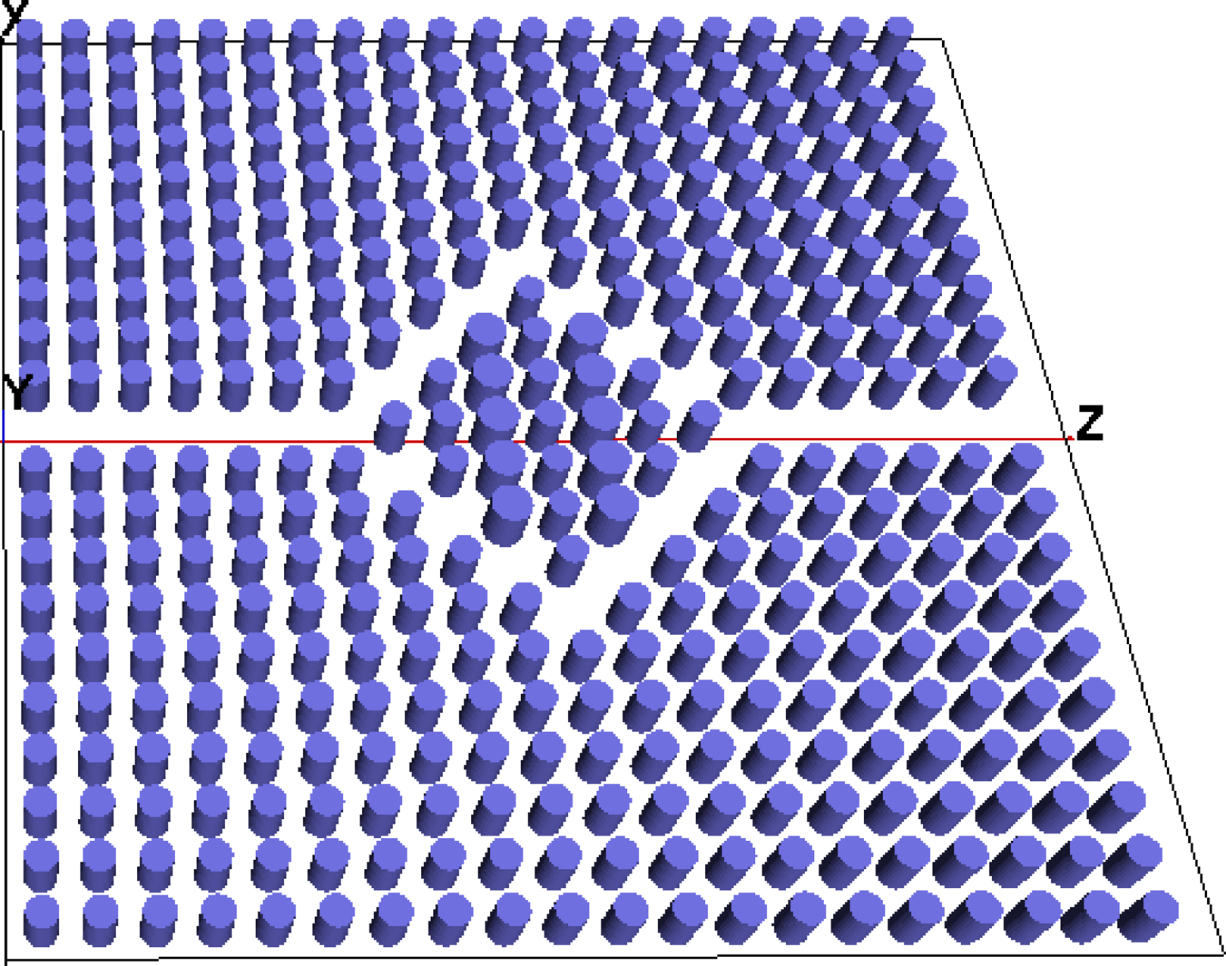
3D model layout.

Figure 4 depicts refractive index profile of the proposed sensor. Two sensing regions are arranged in between the photonic crystals. On applying an analyte with low refractive index, there occurs a dip in refractive index profile in the middle region. The PC refractive index is high, depicted in red color. The Fig. 5 illustrates three dimensional layout of sensor design. The photonic crystal rods are illustrated as light blue color in the diagram. The 3D model helps us to get an overall visualization of sensor topology giving insights into the realistic appearance of sensor.

### Sensor metrics

Various sensor metrics which can be analysed are : wavelength sensitivity, quality factor (QF), detection limit (DL), figure of merit (FOM), spectral width and resolution.

####  Wavelength sensitivity^59^

When the material refractive index changes, there occurs a corresponding change in optical wavelength of output signal. It gives insight into changes that occur in the refractive index (RI) of the sensing region that can be detected.

4 $$S={\text{ }}\frac{{\Delta \lambda }}{{\Delta n}}~\left( {{\text{nm/RIU}}} \right)$$

####  Quality factor (QF)^59^

It gives a perspective on how the sensor confines light within the photonic crystal structure. QF can be calculated by taking the fraction of resonant optical wavelength and spectral width of signal detected at observation point.

5 $$Q={\text{ }}\frac{{\lambda_{resonant}}}{{ {\Delta \lambda _{FWHM}}}}$$

####  Detection limit (DL)^59^

It signifies proficiency in detecting minimum change in index of refraction. Or it may be characterized as the smallest physical change which can be detected by a sensor.

6 $$DL={\text{ }}\frac{\lambda }{{10SQ}}{\text{}}\left( {{\text{RIU}}} \right)$$

####  Figure of merit (FOM)^60^

FOM demonstrates how much the sensor is reliable and sensitive.

7 $$FOM={\text{ }}\frac{S}{{FWHM}}~{\text{(RI}}{{\text{U}}^{{\text{-}}\,{\text{1}}}}{\text{)}}$$

####  FWHM^59^

The difference between the wavelength values corresponding to the two specific half maximum points gives the FWHM.

#### Resolution

It denotes the smallest shift in the wavelength that can be measured with accuracy^61^.

### Novelty and relevance of GaN PC sensor

The novelty of this study lies in its integrative approach to skin diagnostics related to cutis laxa and vitiligo, combining use of GaN, biological relevance, and ML. Two different values of rod radius in a specific two row pattern is used in this work, which is unique as per our knowledge. This adds novelty to the design while maintaining satisfactory performance. Skin tissue classification is a critical step in dermatological diagnostics, and our design targets subtle refractive index differences among tissue types such as dermis, epidermis, and collagen. The use of 2D PC structures tailored to detect elastin, offers a targeted pathway for assessing connective tissue integrity. This is particularly significant in the context of cutis laxa, a rare disorder characterized by abnormal elastin degradation and skin laxity. Disorder such as cutis laxa is rarely studied using 2D PC and especially using GaN along with ML. All these factors makes this 2D PC work a novel one. By using materials such as GaN to enhance optical sensitivity, the design enables precise spectral interrogation of skin biomarkers. Furthermore, the incorporation of ML algorithms into the analysis allows for robust classification of skin pigments facilitating detection and differentiation of pathological skin conditions. Cross validation is also done in this work, which displays the ability of the classifier to classify skin pigments even in noisy conditions.

## Results and discussion on the gallium nitride based PC sensor

**Fig. 6:**
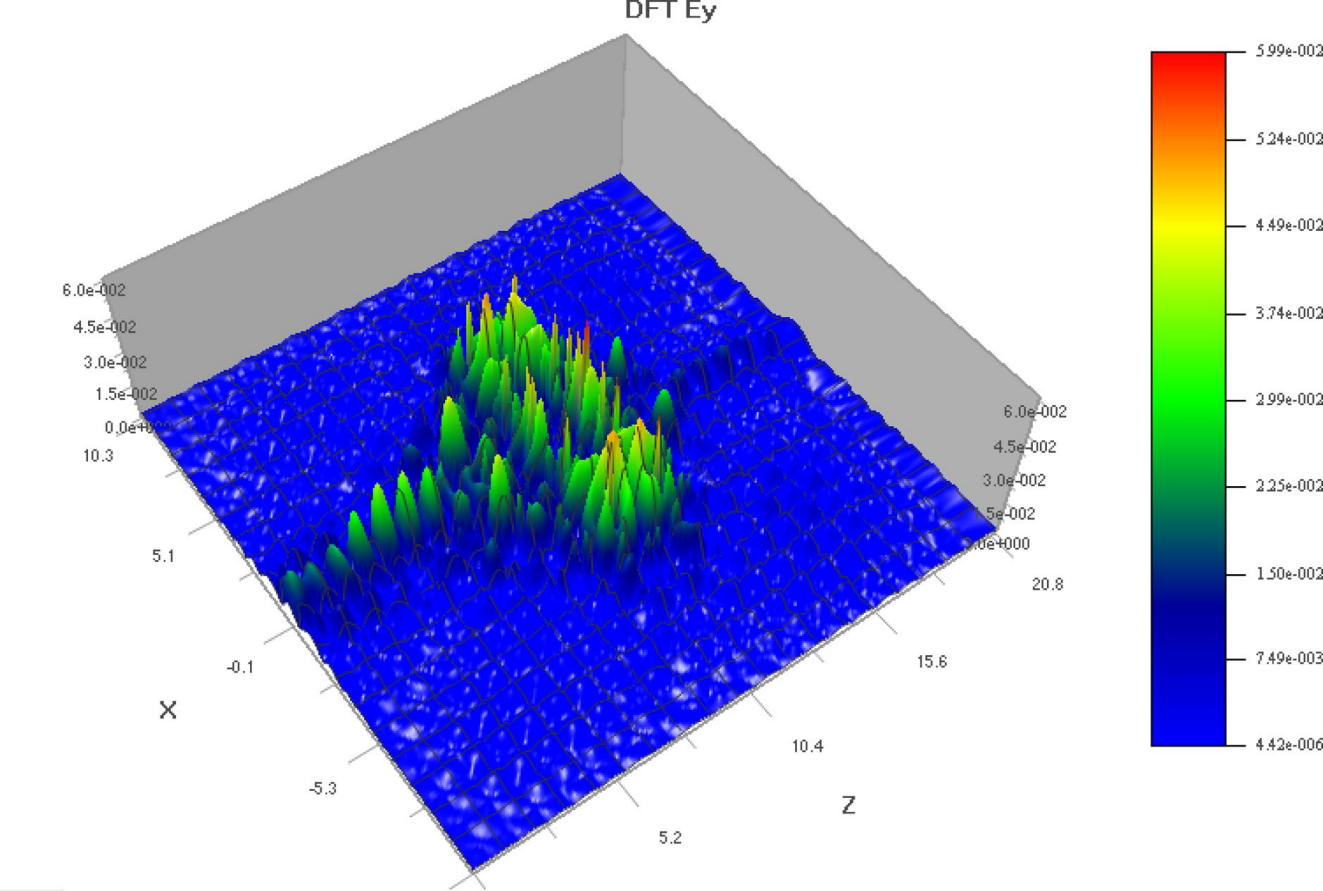
E_y_ plot of the sensor (top side view).

Figure 6 shows the field distribution of the signal propagating through the sensor area which was computed using Discrete Fourier Transform (DFT). The E_y_ plot helps us to understand how the wave propagates through the sensor.

In 2D PC simulations, E_y_ often governs the transverse electric (TE) mode behavior and helps visualize field confinement and resonance near defect sites. Figure 6 helps in TE mode analysis, to understand energy distribution and modal symmetry in the structure. Poynting vector (PV) describes the directional energy flux of the electromagnetic wave. In simulations, it reveals how energy propagates through the photonic crystal, especially near defects or interfaces. The magnitude of the vector indicates power density, while its direction shows the flow of energy. PV visualize energy flow and confirm directional power confinement within the defect region of the PC. Unlike field intensity plots, the PV provides insight into mode propagation and localization, which is critical for validating the sensor’s ability to concentrate energy near the analyte region. Physical principles of propagation in PC waveguides and understanding the losses are also essential in the design^62^.

**Fig. 7:**
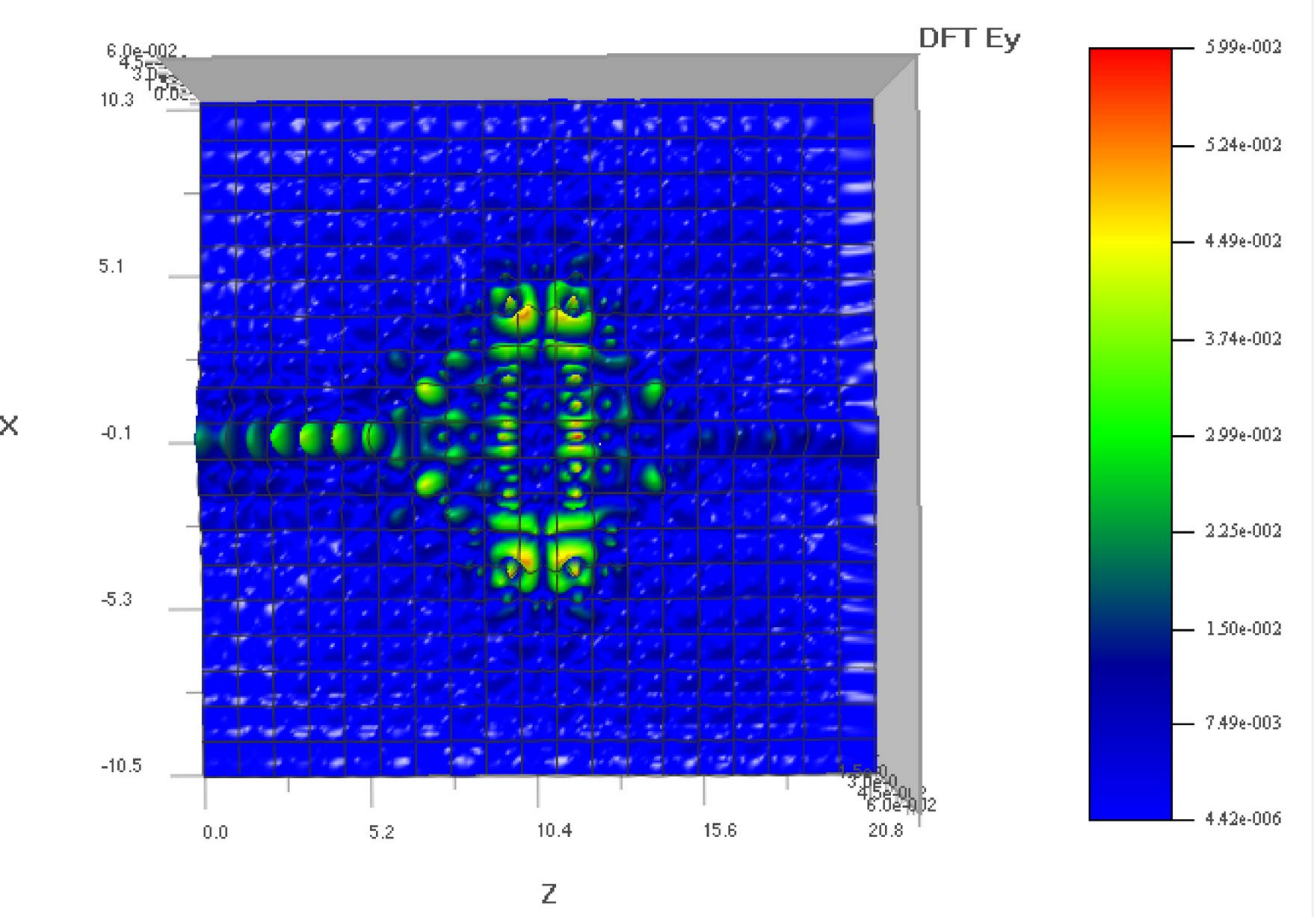
E_y_ plot of the sensor (top view).

**Fig. 8:**
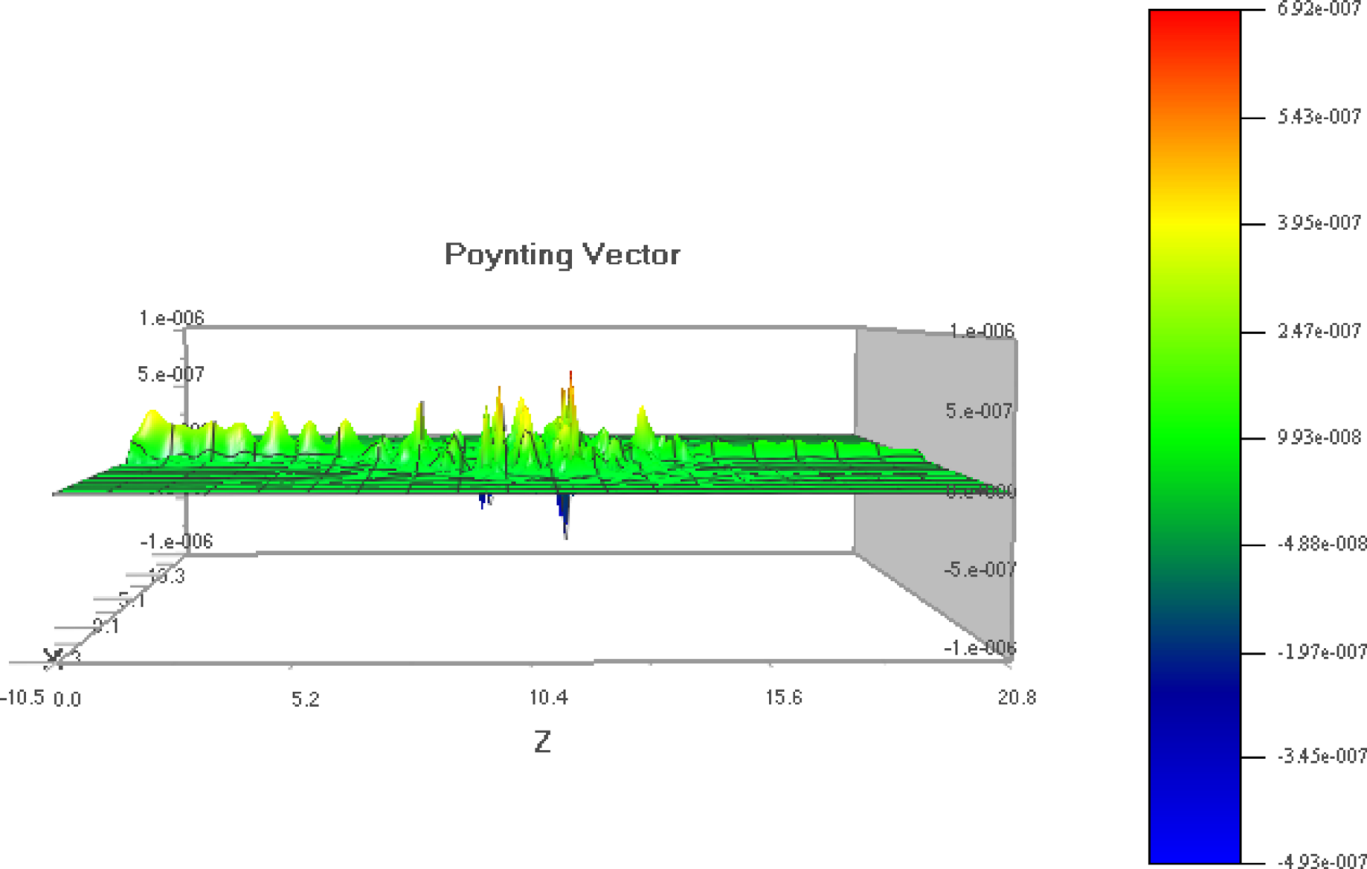
Poynting vector diagram.

The top view of the E_y_ component is shown in Fig. 7 and the pointing vector diagram is shown in Fig. 8. The poynting vector diagram shows the propagation of electromagnetic field over the sensor surface.

**Fig. 9:**
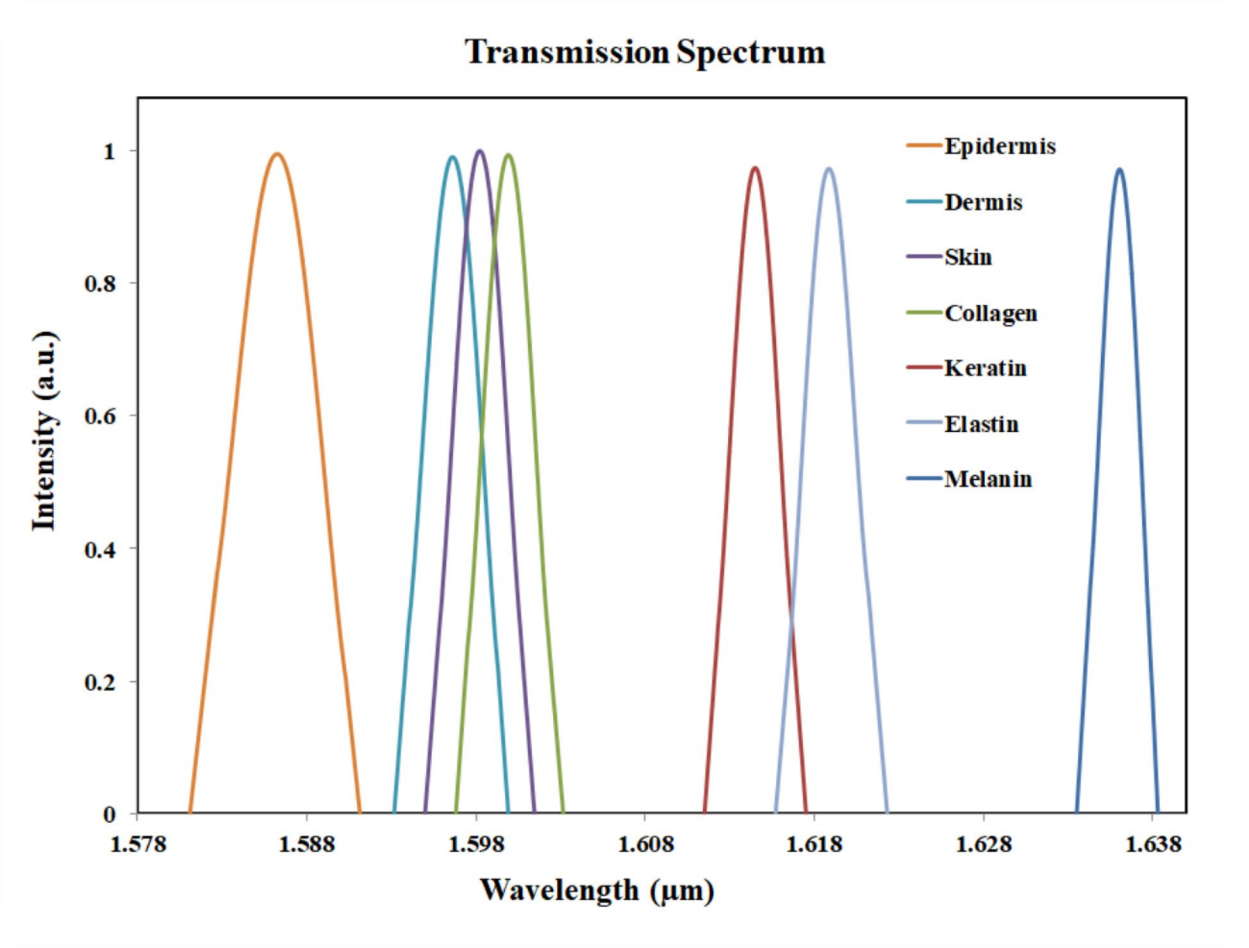
Relative transmission profile depicting various analytes.

The main result of the simulation is the transmission spectrum. The transmission spectrum displays the signal intensities of different analytes under investigation. PC attained a maximum value of 208 nm/RIU and a minimum value of 91 nm/RIU for wavelength sensitivity. The results shown in the Fig. 9 provides the comparison of the various analytes such as melanin, keratin, collagen, epidermis, dermis, elastin and skin. From the wavelength vs. refractive index graph, slope was calculated and the sensitivity is found as 134.96 nm/RIU.

The shift from operating wavelength is caused due to the specific PC design parameters-namely, the lattice constant and rod radius, which were optimized to achieve high-Q resonance and analyte discrimination. The defect-induced resonance modes naturally shifted toward longer wavelengths due to the high refractive index contrast and mode confinement in GaN.

The peak values of the results can be used to identify the various analytes. The pigments which have higher refractive index are observed to have a shift towards the right in the spectrum. The epidermis has peak value at 1586 nm. The dermis, skin and collagen got its peak value at 1596 nm, 1598 nm and 1600 nm respectively. Keratin was noted to have peak value at 1614 nm. Melanin is having a higher refractive index and its peak value is found to be at a wavelength of 1636 nm. Peak value of Elastin is at 1619 nm and it obtained the highest sensitivity. Consequently, the transmission profile of PC sensor helps to distinguish between the various analytes.

**Fig. 10:**
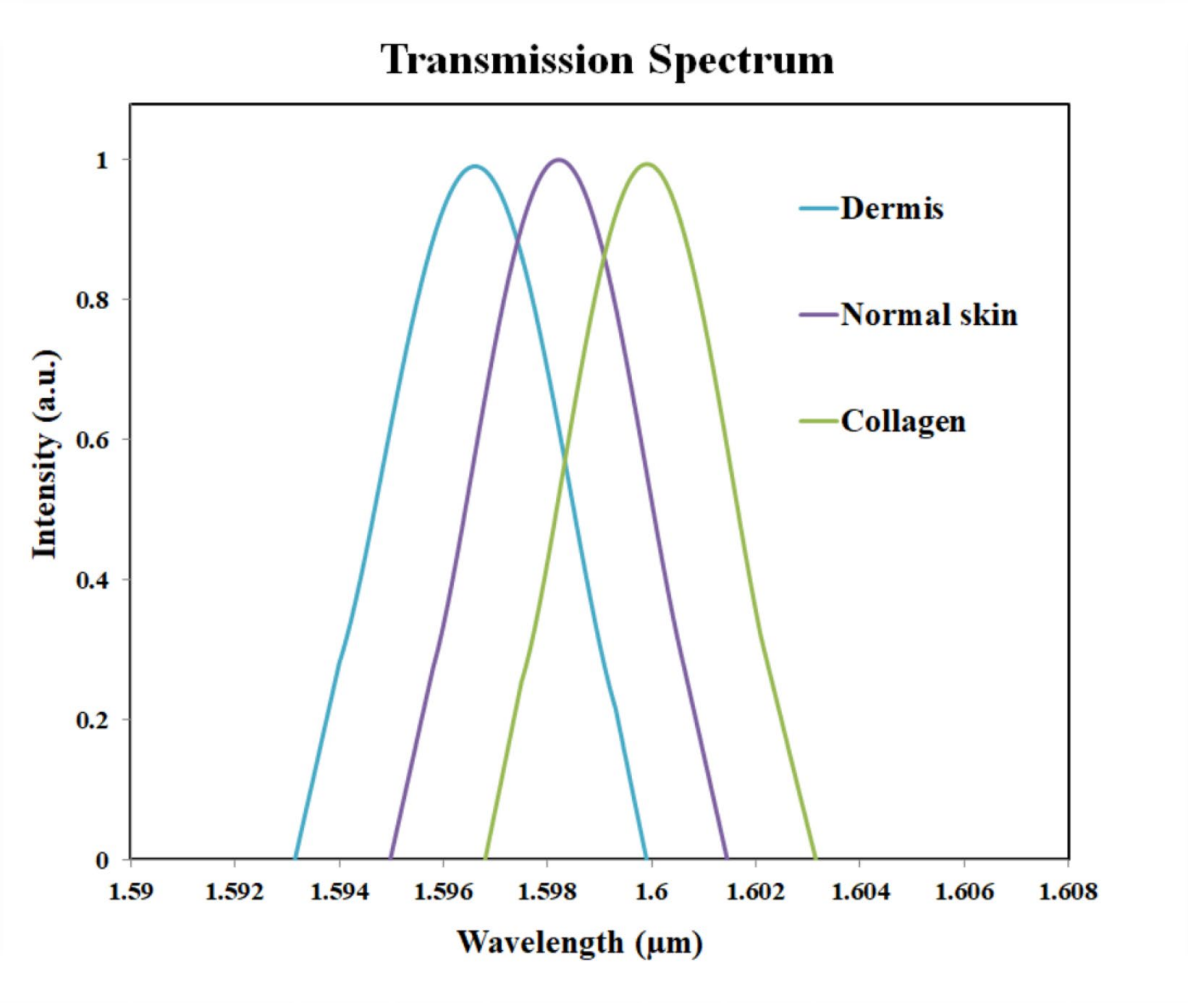
Transmission plot for collagen, skin and dermis.

**Fig. 11:**
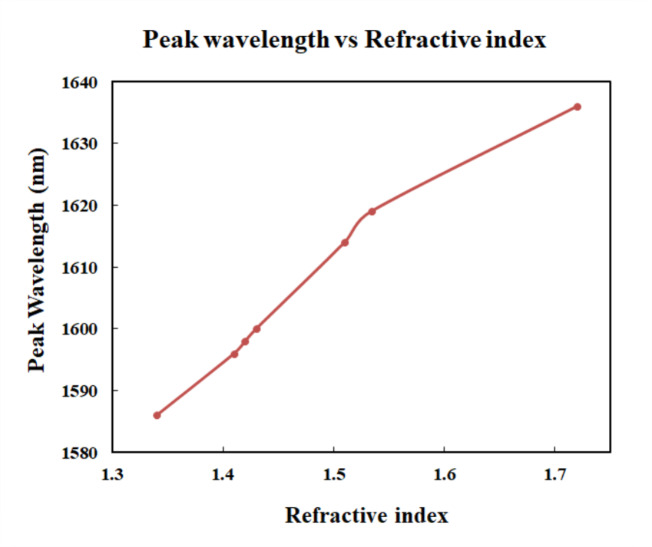
Plot of Peak wavelength vs. of refractive index.

Figure 10 shows the comparison of the pigments which have refractive index values very closer to each other. Although the curves for the collagen and dermis are close to the curve of skin, there is difference in the peak value for each. Thus, the three closer analytes can be identified. Mode confinement, closer refractive index values and defect-induced shifts can be the reasons for closer plots in Fig. 10. The Fig. 11 shows the variations in peak wavelength with respect to the changes in refractive index. For a higher refractive index analyte, the peak wavelength was shifted to 1636 nm. And for a lower refractive index analyte, the wavelength shift is observed at 1586 nm.

**Fig. 12:**
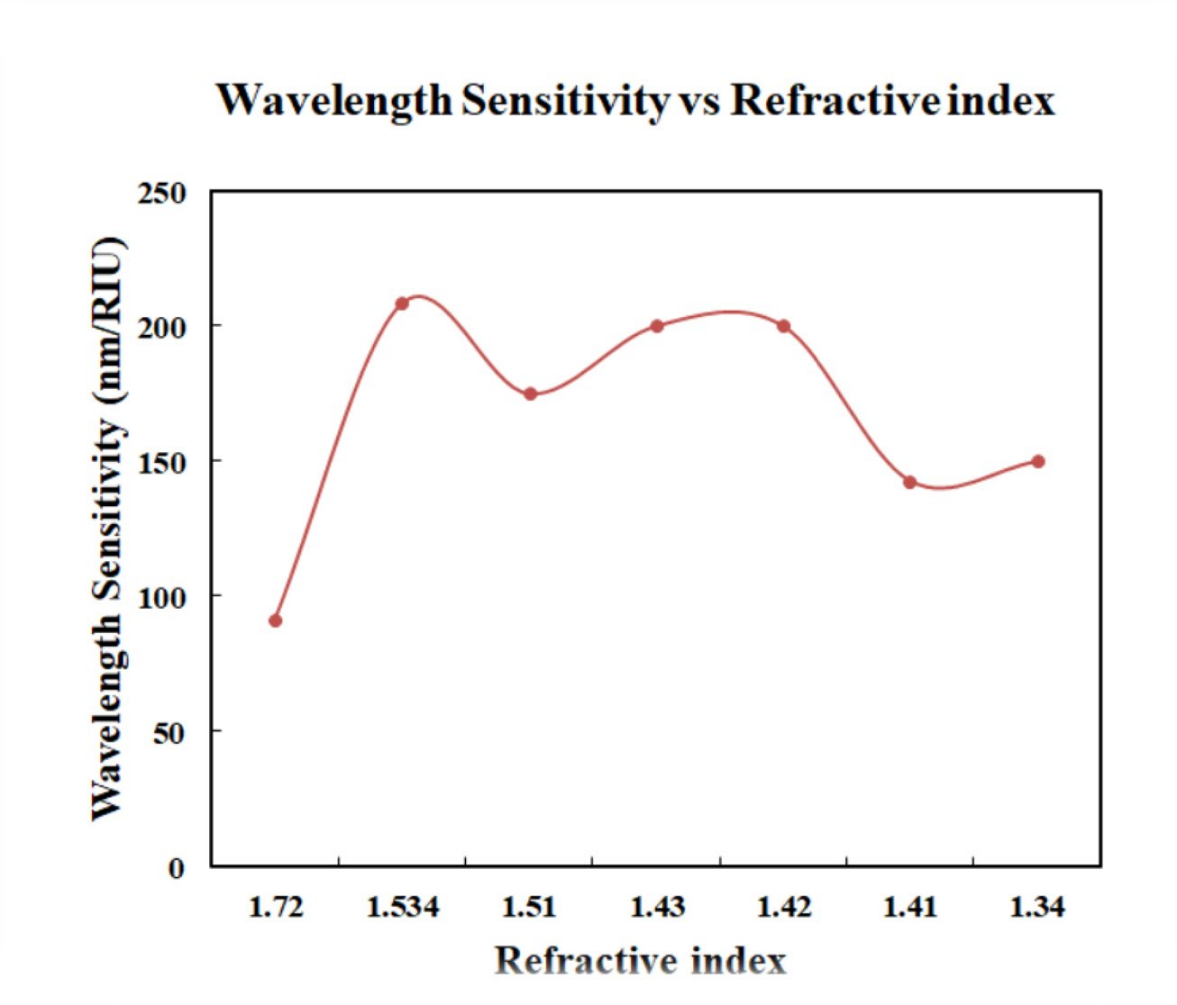
Plot of Wavelength sensitivity vs. of refractive index.

The Fig. 12 shows the variation in wavelength sensitivity for different values of refractive index. The highest sensitivity was achieved for refractive index value of 1.534. The sensitivity variation may arises due to localized defect modes and nonlinear dispersion effects within the GaN PC structure. These effects cause the resonance shift to deviate slightly from linearity across the RI range, especially near high-index analytes. While sensitivity is often treated as constant in idealized models, real-world structures - especially those with engineered defects, can exhibit refractive index dependent sensitivity.

**Fig. 13:**
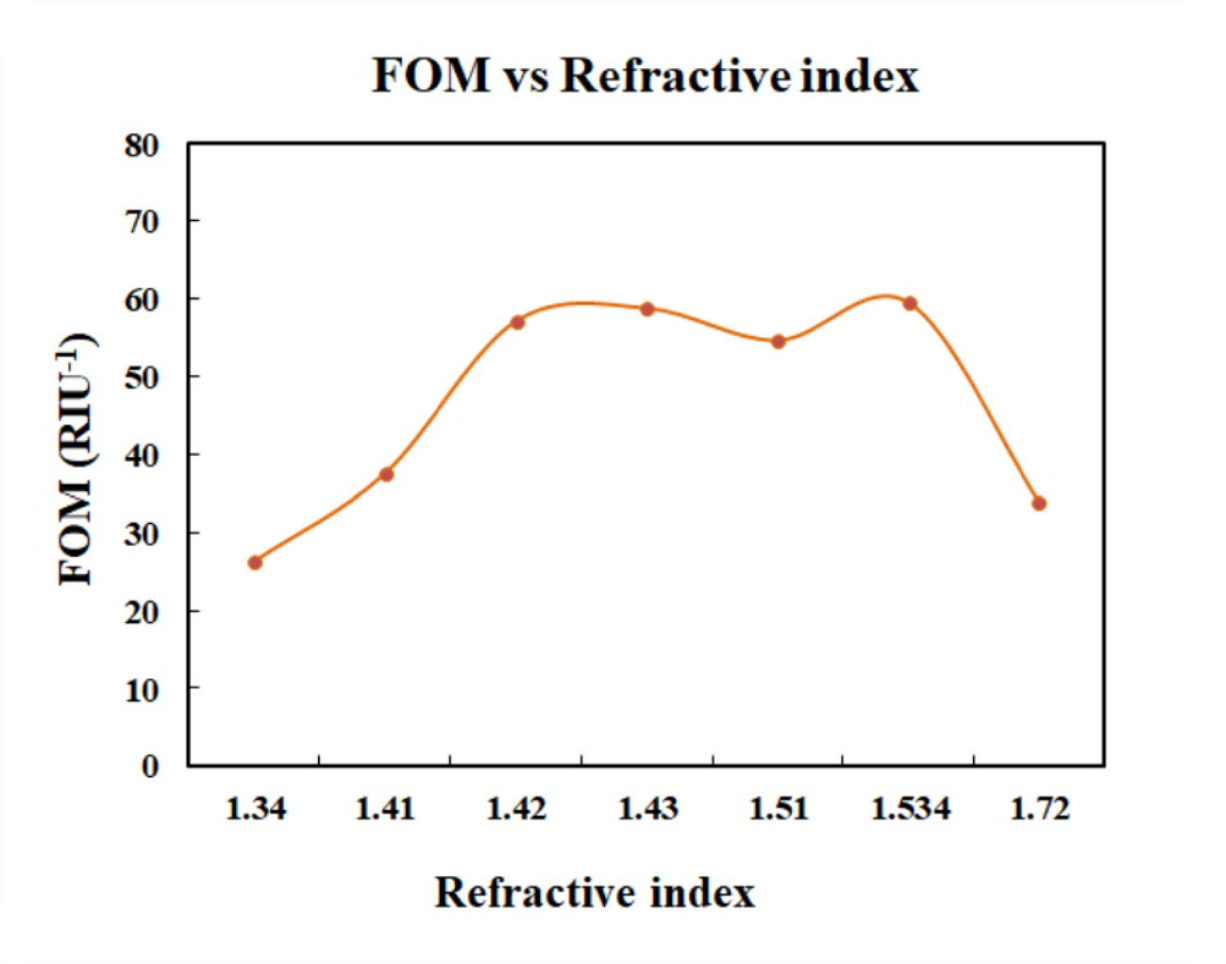
Plot of FOM vs. refractive index.

**Fig. 14:**
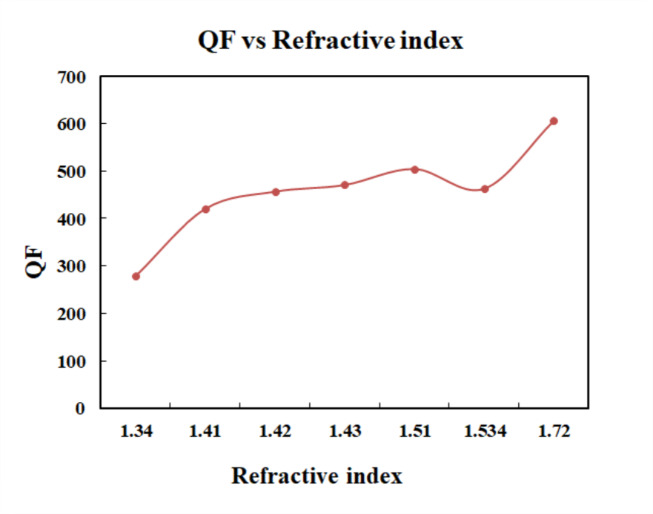
Plot of QF vs. refractive index.

**Fig. 15:**
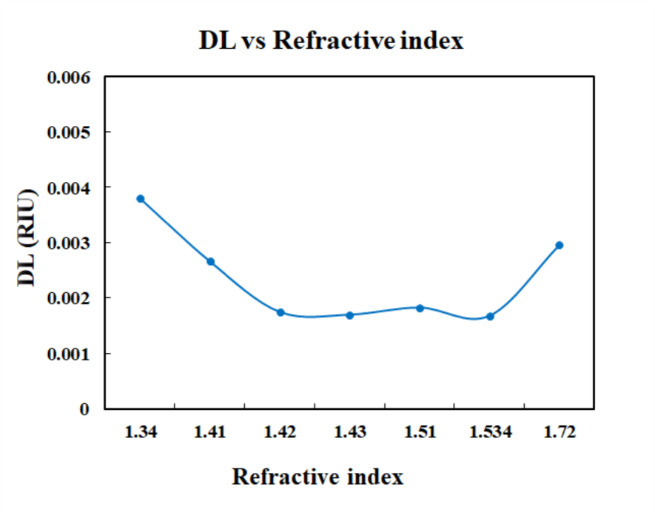
Plot of DL vs. refractive index.

The Fig. 13 shows the plot of FOM and index of refraction. The sensor achieved the highest value of FOM for the refractive index of 1.534. The Fig. 14 shows the plot between QF and refractive index values of the analytes. The highest QF is achieved for refractive index of 1.72. Thus, the sensor provides a better quality factor for melanin. Figure 15 displays the variation of detection limit with respect to refractive index.

The Table 1 shows the various parameters of the sensor for each analyte. Optical wavelength shift with respect to input light is 86 nm for melanin and is the longest transition in this plot. Values of index of refraction for various pigments are also tabulated in Table 1, with normal skin refractive index being taken as the reference for other analytes^63,64^. The global sensitivity achieved by the sensor for refractive index values from 1.34 to 1.72 is 131.58 nm/RIU. And the average sensitivity attained by the sensor is 166.79 nm/RIU.

**Table 1:** Parameters of the gallium nitride based PC Biosensor.

	Refractive index	Peak wavelength (nm)	Wavelength sensitivity (nm/RIU)	FWHM (nm)	QF	DL (RIU)	FOM (RIU^− 1^)
Melanin	1.72	1636	91	2.7	605	0.00295	33.9
Elastin	1.534	1619	208	3.5	462	0.00168	59.5
Keratin	1.51	1614	175	3.2	504	0.00183	54.7
Collagen	1.43	1600	200	3.4	470	0.00170	58.8
Skin	1.42	1598	200	3.5	456	0.00175	57.1
Dermis	1.41	1596	143	3.8	420	0.00266	37.6
Epidermis	1.34	1586	150	5.7	278	0.00380	26.3

### Analysis of the skin diseases

The analysis of the vitiligo disease can be done with the help of transmission profile findings. Initially, samples of normal skin can be applied to sensor area and the transmission spectrum can be analysed. The vitiligo infected cells can be applied on the sensor to study the shift in the wavelength. Thus, by the comparison of the transmission spectrum of both cases, the differences are recognized. Optical wavelength shift is distinguishable between normal skin cells and vitiligo affected skin cells which in turn helps us to identify each of them. The comparison plot of normal skin with collagen and dermis pigmentation shown in Fig. 10 helps to distinguish the normal skin from the collagen and dermis pigmentation.

**Fig. 16:**
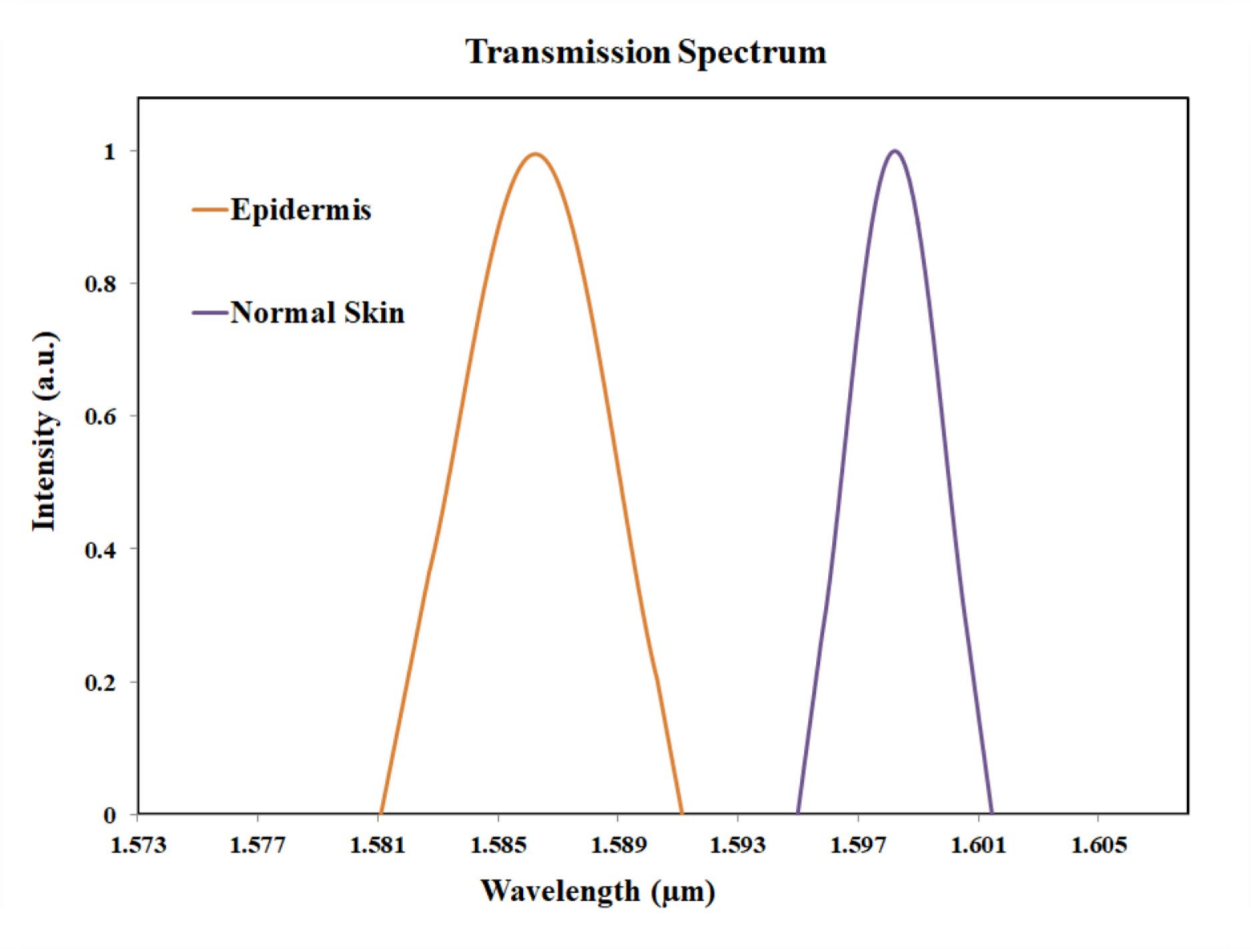
Transmission plot for normal skin and epidermis pigmentation.

The difference in the peak value between the normal skin cells and collagen cells are 2 nm, as the peak value for collagen cells were observed at 1600 nm. The peak value for dermis pigmentation was observed at 1596 nm, and the difference in the peak wavelength compared to the normal skin cells is 2 nm. The Fig. 16 shows the transmission spectrum comparison of normal skin and epidermis pigmentation. The peak value for normal skin cell was at 1598 nm and for epidermis pigmentation, the peak value was observed at 1586 nm. Thus, the shift in wavelength is 12 nm.

**Fig. 17:**
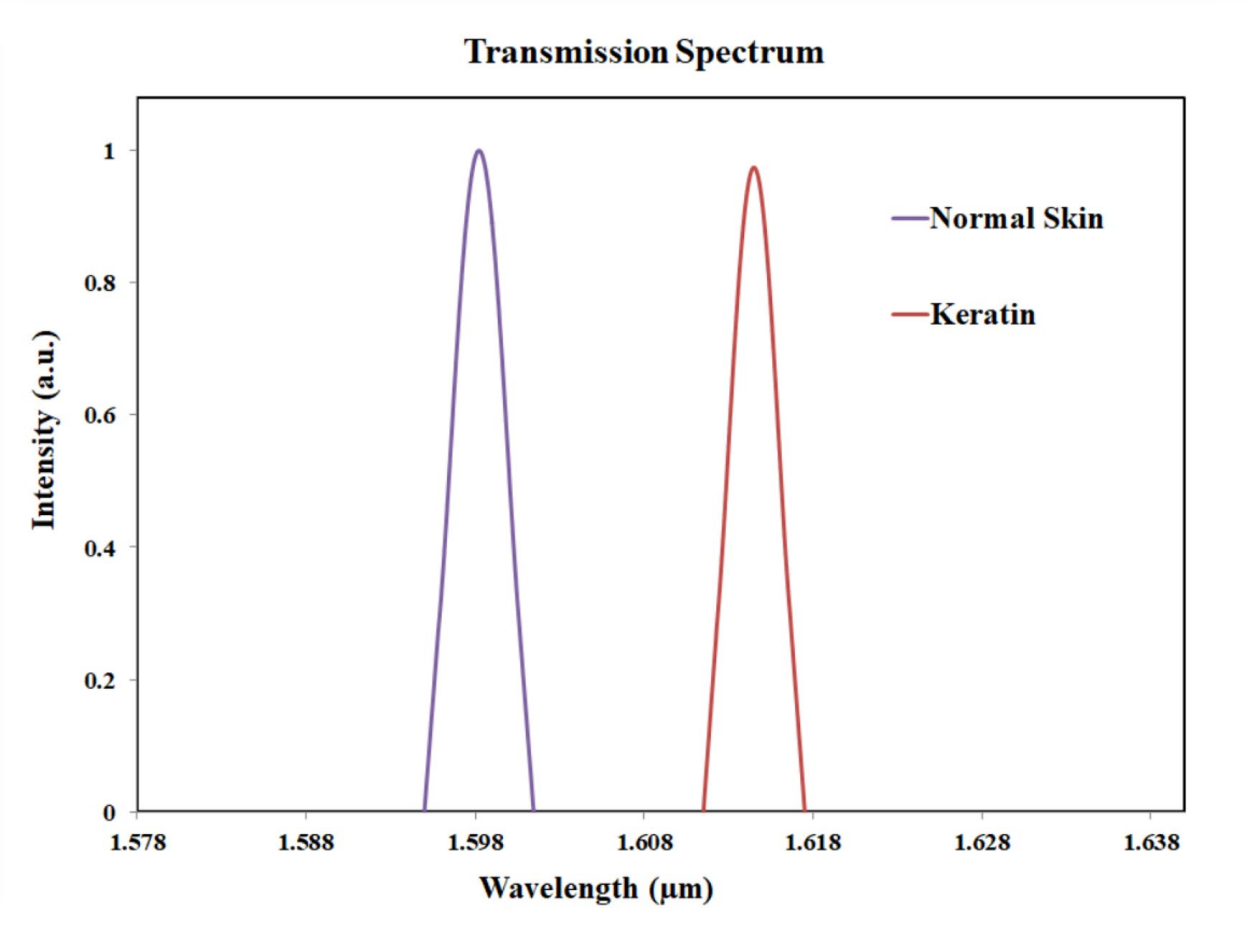
Transmission plot for normal skin and keratin pigmentation.

**Fig. 18:**
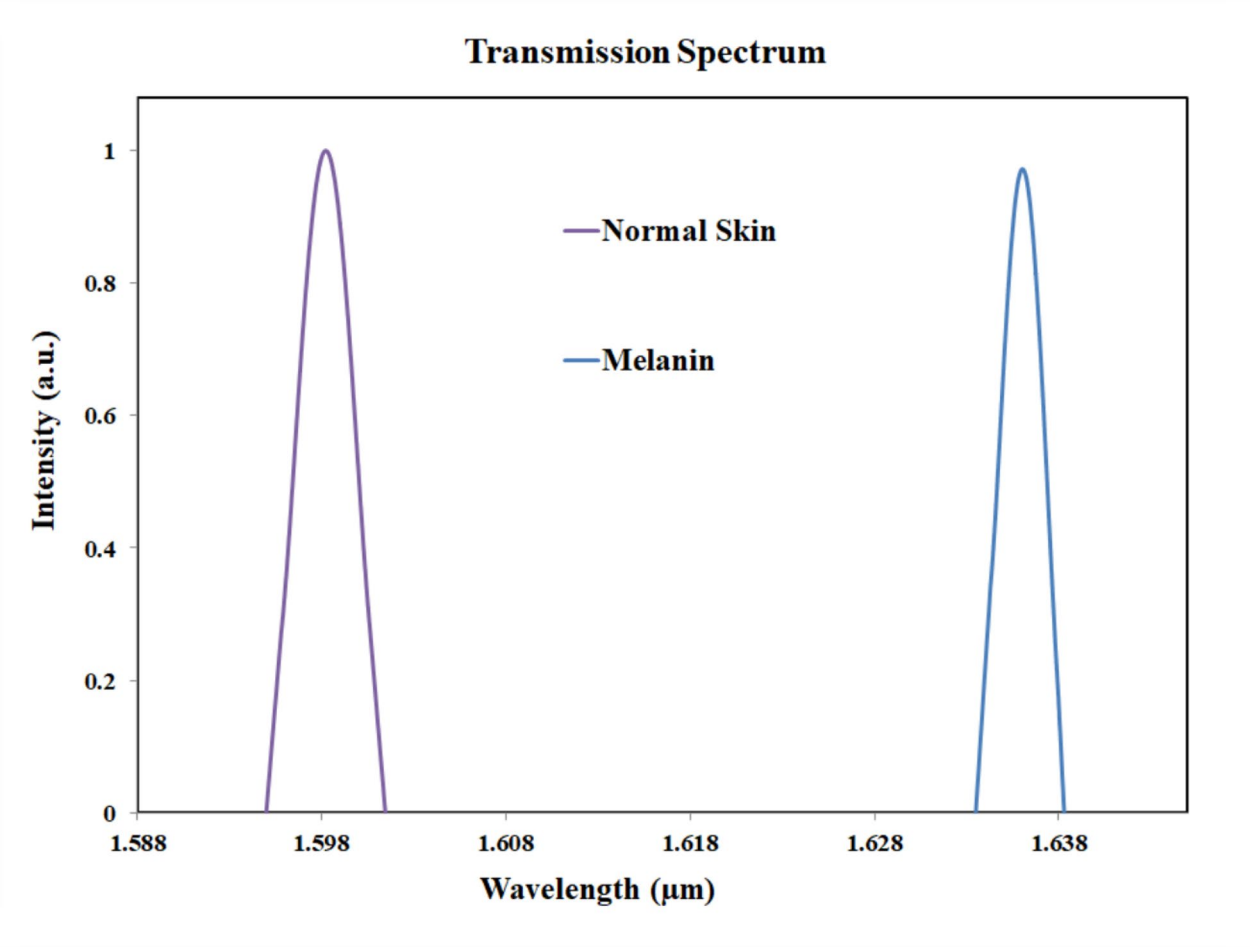
Transmission plot for normal skin and melanin pigmentation.

The Fig. 17 shows the transmission spectrum comparison of normal skin and keratin pigmentation. For keratin pigmentation, the peak value was observed at 1614 nm. Thus, the shift in wavelength is 16 nm. The larger shift in the wavelength can differentiate normal skin from keratin pigmentation. The Fig. 18 shows the transmission spectrum comparison of normal skin and melanin pigmentation. The peak value melanin pigmentation was observed at 1636 nm. Thus, the shift in wavelength is 38 nm. The larger shift in the wavelength differentiates normal skin from melanin pigmentation. Early detection of vitiligo and cutis laxa is clinically valuable, especially in pediatric and geriatric populations. Our biosensor targets subtle refractive index changes associated with tissue degeneration, offering a non-invasive and potentially real-time diagnostic tool.

From the results of the photonic crystal sensor data, we can understand the variations in the output of the sensor when different pigmentations are applied to the sensor. As each pigment has a particular refractive index, the sensor produces a corresponding shift in the output light wavelength. This in turns helps us to distinguish the various skin pigments under investigation.

**Fig. 19:**
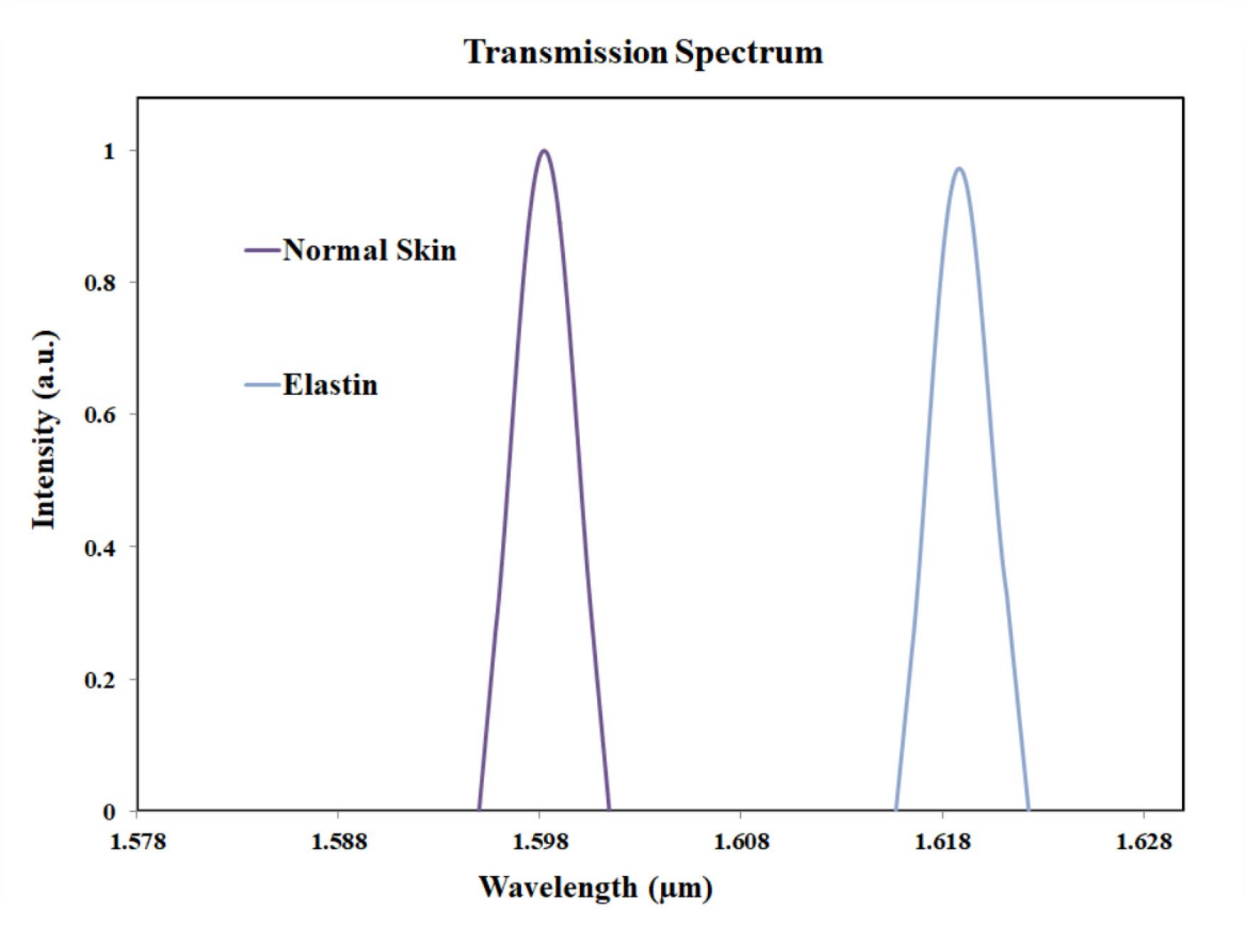
Transmission plot for normal skin and elastin.

Comparison of skin and elastin is shown in Fig. 19. The peak value for elastin was observed at 1619 nm. Thus, the shift in wavelength with respect to normal skin sample is 21 nm. Skin disease such as cutis laxa may be identified by comparing the normal skin and the variation in elastin.

The Table 2 shows the relative assessment of sensitivity and QF of GaN based sensor with the preceding investigations in biosensing using 2D PCs. The GaN based sensor secured a notable value of 208 nm/RIU for its sensitivity compared to the results of earlier works of others. GaN based devices are promising in the field of optical sensors^74^.

Table 3 displays numerous studies related to photonics and throws light on the relevance of current work in the domain. Table 4 showcases the comparison of GaN with other materials which are used in PC based sensors.

**Table 2:** Comparison of sensitivity results with earlier work of others.

Reference	Year	Sensing approach	Analytes/disorders investigated	Sensitivity (nm/RIU)	QF
Ref^65^	2019	2D PC	Blood components	--	262
Ref^66^	2022	2D PC	Blood components	2.9	5166
Ref^67^	2024	2D PC	Proteinuria detection	110	15,466
Ref^55^	2024	2D PC	Blood cholesterol	32	4988
Ref^68^	2024	2D PC	Protein	100	597
Ref^69^	2024	2D PC	Alcohol	--	--
Ref^70^	2024	2D PC	Skin cancer	72	1741
Ref^71^	2024	2D PC	Cancer cells	55	14,780
Ref^72^	2025	2D PC	Brain tissue	79	31
Ref^73^	2025	Multilayer PC	Skin cancer	378	67
Proposed work	2025	2D PC	Elastin, keratin, melanin, collagen, skin, dermis, epidermis	208	605

**Table 3:** Comparative insights into simulation studies related to photonic applications.

Year	Topic of research	Material/Structure	Application Domain	Methodology	Ref
2012	Strength of Cygel via 2D PC	2D PC	Biomedical gel	Simulation based on plane wave expansion (PWE)	^75^
2013	Potassium chloride Sensor Using PCF	Photonic Crystal Fiber (PCF)	Compound detection	Simulation based on plane wave expansion (PWE)	^76^
2014	Temperature in Semiconductor using optical principle	Compound semiconductor	Thermal	PWE simulation	^77^
2015	Glucose in Intralipid	Microstructured fiber	Tissue diagnostics	PWE method	^78^
2016	Mole Fraction in Nitride	Nitride semiconductor	Material analysis	Photonic Bandgap analysis	^79^
2016	Studies on temperature variation	Semiconductor waveguide	Nanophotonics	Loss analysis and PWE simulation	^80^
2018	Optical loss studies in Waveguide	Semiconductor waveguide	Estimation of Scattering and reflection losses	Simulation using transfer matrix method	^81^
2019	DNA/Protein Estimation	2D Photonic structure	Estimation of concentration of proteins	Simulation using PWE	^82^
2019	Angular Waveguide Sensor	Photonic waveguide	General sensing	FDTD simulation	^83^
2020	Refractive Index of Viruses	Zirconium nanoparticle	Photo luminescence intensity based studies	Reflectance analysis	^84^
2021	Pressure Sensor	Germanium photonic structure	Pressure sensing	Numerical simulation	^85^
2023	Bacterial Water Detection	Ring resonator	Water quality	FDTD simulation	^86^
2023	Detection of water content using He-Ne Laser	1D Photonic Waveguide	Assesment of water in biological tissue	PWE simulation	^87^
2024	Sensor for investigation of heavy metal	Photonic Crystal	Industrial water studies	PWE simulation	^88^
(2025) This work	Detection of skin related analytes	2D PC	Biosensing	FDTD simulation and ML	--

**Table 4:** Comparative study of GaN with silicon, silicon nitride and titanium dioxide.

	GaN	Silicon	Silicon Nitride	Titanium dioxide
Bandgap (eV)	~ 3.4^57^	~ 1.12^70^	~ 5.1^89^	~ 3.0–3.2^90^
Refractive index	~ 2.25–2.5^56^	~ 3.4–3.5^91^	~ 1.99–2.0^92^	~ 2.4–2.92^93^
Transparency Range	UV to visible	Near-IR to visible	Visible to near-IR	UV to visible
Biocompatibility	High (non-toxic, stable)	Moderate	Excellent	Excellent
Thermal Stability	Excellent	Good	Excellent	Moderate
Fabrication Maturity	Emerging	Very mature	Mature	Less mature for integrated photonics
Nonlinear Optical Properties	Strong	Weak nonlinearities	Low nonlinear response	Good for nonlinear optics
Photonic Crystal Suitability	High-Q, UV-visible operation	High-Q, IR-visible operation	Low-loss waveguides, visible range	High-index contrast, UV-visible
Surface Chemistry Tunability	Moderate	High	Moderate	High
Integration with ML Systems	Compatible with hybrid platforms	Fully compatible	Compatible	Emerging use in ML-enhanced sensors

The advantage of defect-localized resonance in PC over conventional techniques like scattering or absorption spectroscopy is that it offers enhanced spectral selectivity, noise suppression, spatial localization and compact integration.

## Incorporating machine learning for the classification of analytes

Artificial intelligence (AI) is operationalized in PC biosensor as it offers a large number of assets. It can implement processes and functionalities that may take more time for a human to accomplish. Primary motive to include machine learning (ML) in PC sensor system is to fine tune accuracy in classifying the data points such as the peak wavelength of the output signal from the photonic crystal.

Supervised and unsupervised learning techniques can be used for predicting the presence or absence of a disease. ML algorithms are leveraged for learning the patterns of the spectral values and predicting the output. Different models are used for prediction of diseases. The commonly used models are logistic regression, support vector machine, Random forest (RF), artificial neural network (ANN) and Naive Bayes^94^. RF and SVM models may be deployed for the detection and classification of skin samples affected by vitiligo. The Random Forest Model exhibits better accuracy than SVM^95^. The various samples of the skin have different refractive index values. The spectral width, optical wavelength shift of signal and output signal intensity from the sensor is influenced by the analyte refractive index. ML models can be used to classify the vitiligo pigments based on the peak value of the output signal from PC. Data collected from PC sensor simulation includes wavelength, FWHM and intensity of light. This data can be used as the input to the machine learning model.

ML can be integrated with two-dimensional PCs to find concentration of analytes with accuracy. Nonlinear feature association between optical sensor signal and analyte concentration can also be modeled using long short-term memory (LSTM) algorithm. The precision in detection is enhanced by ML^69^. The dataset for ML can also be the output spectrum of the sensor and corresponding wavelength. KNN (k-Nearest Neighbor) is another ML which can be used to predict accuracy for the given light energy. KNN can also be used for Multi-class classification. The data points which are closer to each other can be classified using KNN. Thus, in the case of Vitiligo, KNN can be used to classify the datasets of collagen, skin and epidermis which have refractive indices very close to each other. By calculating the Euclidean distance, the k-nearest neighbors can be identified. Thus, the various analytes can be classified. By using a majority voting method, the class of analyte under investigation can be found. The test value is assigned with the class label that is common to the k neighbours. Classification and Regression Trees (CART), SVM and ANN are other efficient ML techniques which provided accuracies of and 93.49%, 90.5% and 86.3% respectively for the detection and identification of diseases. Fuzzy Logic, Logistic Regression, XG Boost algorithm and XGB Classifier algorithms can also achieve higher accuracies. Compared to these algorithms, KNN algorithm can provide an accuracy of 97.12%^96^.

The KNN method is effective for identifying the various types of skin variations and thus provides a lot of advantage in the bio sensing. KNN algorithm can be used for classifying a variety of analytes and it would be faster and accurate than the conventional methods. ML algorithms help to do real time analysis and monitoring of the analytes under investigation. Timely assessment of disease and quick analyte identification helps to improve the overall performance of the biosensor. Regarding the computation time, ML training can be completed within seconds per model. Thus, incorporating ML techniques helps to advance the healthcare monitoring.

Multiple regression models can be used in photonic crystal bio sensing. By giving the input to the ML model the dataset such as the data set containing information about the infected cells, the model can be trained. Later the ML model can be tested using another set of data which would contain the parameters such as wavelength and intensity of light which is different from the data which was given during training. Thus, the performance of the ML model can be evaluated with the help of test data. R squared value which gives us the idea about how better the algorithm predicts the outcome can be calculated for the models. The errors in the predicted values of the peak wavelength can be calculated for the multiple regression model and Support Vector Machine model. Compared to multiple regression model, Support Vector Machine model gives better performance in predicting the peak wavelength of the sensor output. The prediction quality of the model can be improved by increasing the data points. The model can be also trained for diverse data, so that it would perform better in identifying a variety of analytes^71^.

The target value can be the peak wavelength or the shift in the wavelength from the transmitted wavelength. The data for testing can be obtained from the FDTD tool. Thus, the model gets trained by giving a set of data of vitiligo infected cells. This training helps the ML model to understand the spectral parameter such as the peak wavelength. Once we give a test data, the model would identify the peak values of the infected cells. Thus, by training the ML model with the available data, the peak value of the wavelength can be identified. ML can be also used to study the relation between the wavelength, FWHM, and the radius of the crystal.

The other approaches done in machine learning were algorithms such as such as Least Squares Regression (LSR) Method, Elastic-Net approach and Bayesian Ridge Regression (BRR) technique. Out of these methods, the least squares method gives a better performance with respect to the prediction of outcomes^97^. The patterns in the transmission spectrum can be recognized using Machine learning techniques. The noisy signal in the spectrum could also be removed by leveraging ML^98^. Training data is used for training the machine learning model with respect to the given data. Testing data is the significant and pivotal data that highlights the functionality metrics of model. Training data for ML strategy will be the data collected after running the simulation using the sensor simulation tool. Thus, the spectral values from sensor output are given to learning algorithm. The output of the algorithm gives the predicted output. Best fit line is selected by comparing the actual data and the predicted output.

**Fig. 20:**
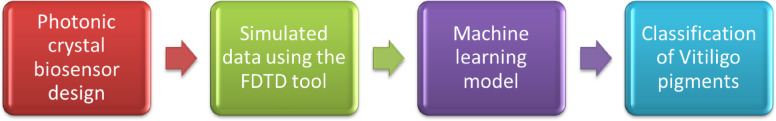
Integrating Machine learning model with the proposed biosensor.

The diagrammatic representation of the integration of machine learning model with the proposed biosensor is as shown in the Fig. 20. The first step in Machine learning is to collect sufficient data for the model. The simulated data is given as the input to the model. Secondly, appropriate algorithm for the model is decided. The third step is to train the model with the available data and the fourth step is to monitor the performance of the model. The various parameters such as accuracy of the model to classify can be evaluated. The last step is to test the model with an unseen data and assess functionality with respect to accuracy of classification. The classification report reveals the computational efficiency and effectiveness of model.

### Flowchart of the proposed machine learning method for classification of skin analytes

**Fig. 21:**
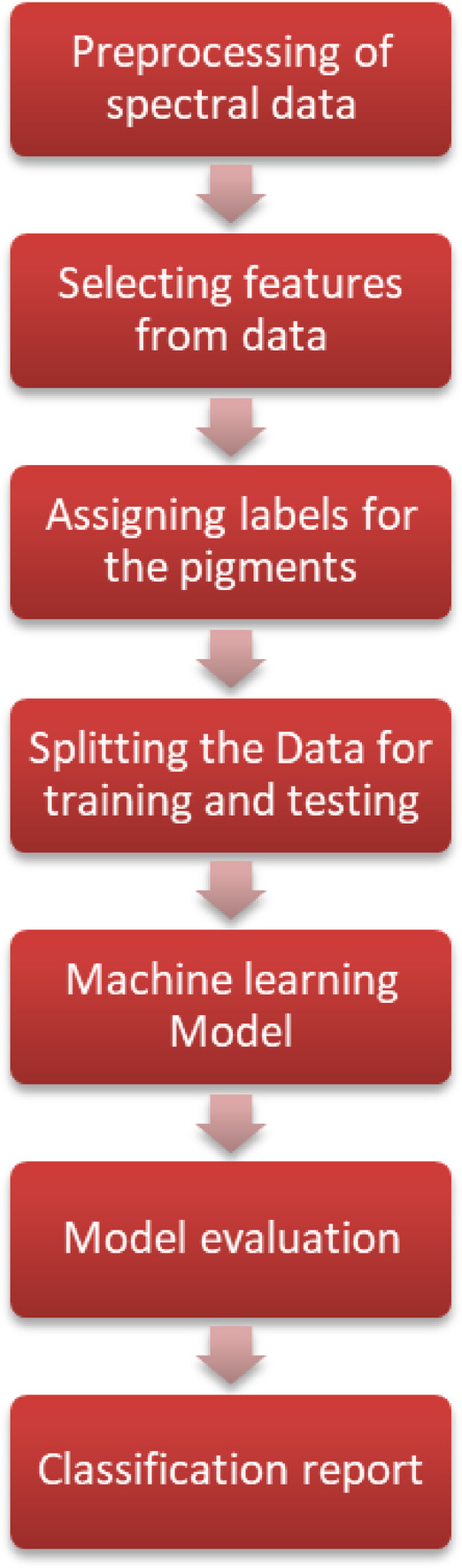
Flow chart for the Machine learning model for classification of skin analytes.

The flowchart for the ML model evaluation is as shown in Fig. 21. Spectral values received from simulation must be preprocessed. The preprocessing is done by assigning class labels such as melanin, keratin, and others under the assumption of equal sample distribution across seven categories. Dataset is trimmed to a size divisible by the number of classes to maintain balance. Features are extracted from the spectral profiles and fed into classifiers. Feature and target separation is performed by removing the label column from the dataset to form the input matrix, while the labels themselves serve as the target vector. Standardization is then applied to the features using z-score normalization, ensuring that each input variable has zero mean and unit variance.

The data (1000 spectra for each analyte) can be normalized for providing consistent features to the model. The second step is selection of the features such as wavelength and intensities of the various analytes. All features present in the dataset (ranging from 1550 nm to 1640 nm) can be used for training. Next step is to assign labels for the respective pigments. The data is split for testing and training purpose. As a standard, 20–30% of the simulated data can be used for testing. The next step involves the application of K-NN, RF, SVM and MLP (Multi-Layer Perceptron) ML algorithms. After evaluation of the model, machine generates report which gives functionality related to classification. Effectiveness of applied algorithms may be interpreted from classification report. The algorithms selected for the classification of analytes were RF, K-NN, MLP and SVM classifier. Machine learning training and testing is done with the help of Google colab platform. Python language code is used for executing the machine learning models. Google colab is one of the web based platform which helps in executing the python language codes. Pandas and NumPy python libraries were used for executing the algorithms.

The RF model is built using biosensor data structured into a feature vector composed of duration, entropy, kurtosis, mean, peak, rms, skewness, slope, standard variance, and zero crossing rate. Each of these descriptors captures a unique aspect of the signal characteristics associated with the seven analytes. Before training, the features are standardized using StandardScaler to ensure uniform scaling across dimensions, which is essential for stable and unbiased model performance. RF is configured with 100 decision trees, each trained using bootstrap sampling to enhance generalization. It uses the Gini impurity criterion to evaluate split quality and allows trees to grow without a predefined depth limit. The model requires a minimum of two samples to split a node and at least one sample to form a leaf. Feature selection at each split follows the default strategy, typically using the square root of the total number of input features. No class weighting is applied, and a fixed random seed ensures reproducibility across runs.

KNN is configured with five neighbors, meaning each prediction is based on the majority vote among the five closest training samples. The model uses uniform weighting, so all selected neighbors contribute equally to the decision. It automatically selects the most efficient algorithm for neighbor search, and the leaf size for tree-based methods is set to 30. The distance metric used is Minkowski with a power parameter of 2, which corresponds to standard Euclidean distance. No parallel processing is specified, and no additional metric parameters are provided. The input feature vector is constructed from biosensor measurements stored in a file, excluding the manually assigned labels for seven analytes. These features are normalized using standard scaling to ensure consistent input ranges across all dimensions.

SVM is configured with a radial basis function (RBF) kernel, enabling it to capture nonlinear patterns in the biosensor data. Key hyperparameters include a regularization parameter C set to 1.0, which balances model complexity and classification accuracy, and gamma set to scale, allowing the kernel coefficient to adapt based on feature variance. The model is trained with probability estimates enabled, using a fixed random state of 42 to ensure reproducibility. Other default settings include unlimited iterations, and no class weighting. The input feature vector is derived from a structured dataset containing biosensor measurements, excluding the manually assigned labels for seven analytes. These features are normalized using standard scaling to ensure consistent input ranges across dimensions.

The architecture of the MLP is composed of two hidden layers with 64 and 32 neurons respectively. Each neuron in these layers uses the ReLU (Rectified Linear Unit) activation function, which introduces non-linearity and helps the network learn complex patterns in the biosensor data. The model is trained using the Adam optimizer, a gradient-based optimization algorithm known for its efficiency and adaptive learning rate. While the current setup does not explicitly include regularization techniques such as L2 penalty or dropout, the iteration parameter acts as a soft constraint on training duration. Early stopping is not enabled in this configuration, meaning the model will continue training until it reaches the maximum number of iterations or convergence. This architecture is well-suited for capturing nonlinear relationships in high-dimensional biosensor features, offering a balance between model complexity and computational efficiency.

### Results of machine learning in the classification of skin analytes

The results after executing the Machine learning algorithms are presented as the classification reports for each of the algorithms namely RF, K- NN, MLP and SVM classifier.

#### Random forest model

Accuracy: 98.80%

**Table 5:** Values secured by Random Forest model.

	Precision	Recall	F1-score	Support
Collagen	1	0.97	0.98	64
Dermis	0.97	1	0.98	57
Elastin	1	1	1	67
Epidermis	1	0.95	0.98	44
Keratin	0.98	1	0.99	55
Melanin	1	0.98	0.99	64
Skin	0.97	1	0.99	67

The classification report gives the idea about the parameters such as accuracy of classification, F1 score, recall value and precision in classification.

**Fig. 22:**
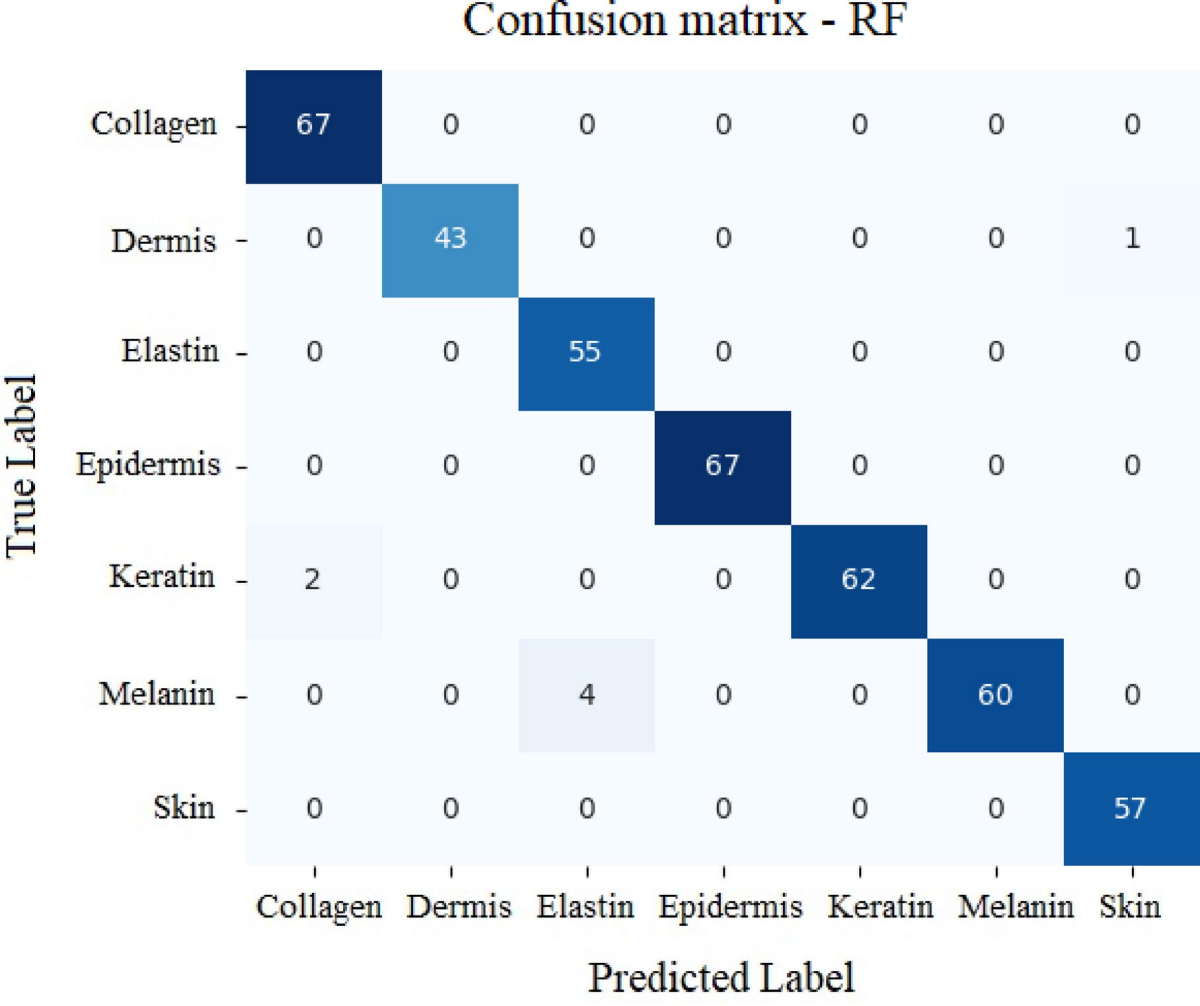
Results for RF – True label vs. Predicted labels.

The RF classifier provided an accuracy of 98.80% to classify various analytes. Table 5 gives the classification report for RF model. Figure 22 depicts results related to true vs. predicted values of analytes.

#### KNN model

Accuracy: 98.56%

**Fig. 23:**
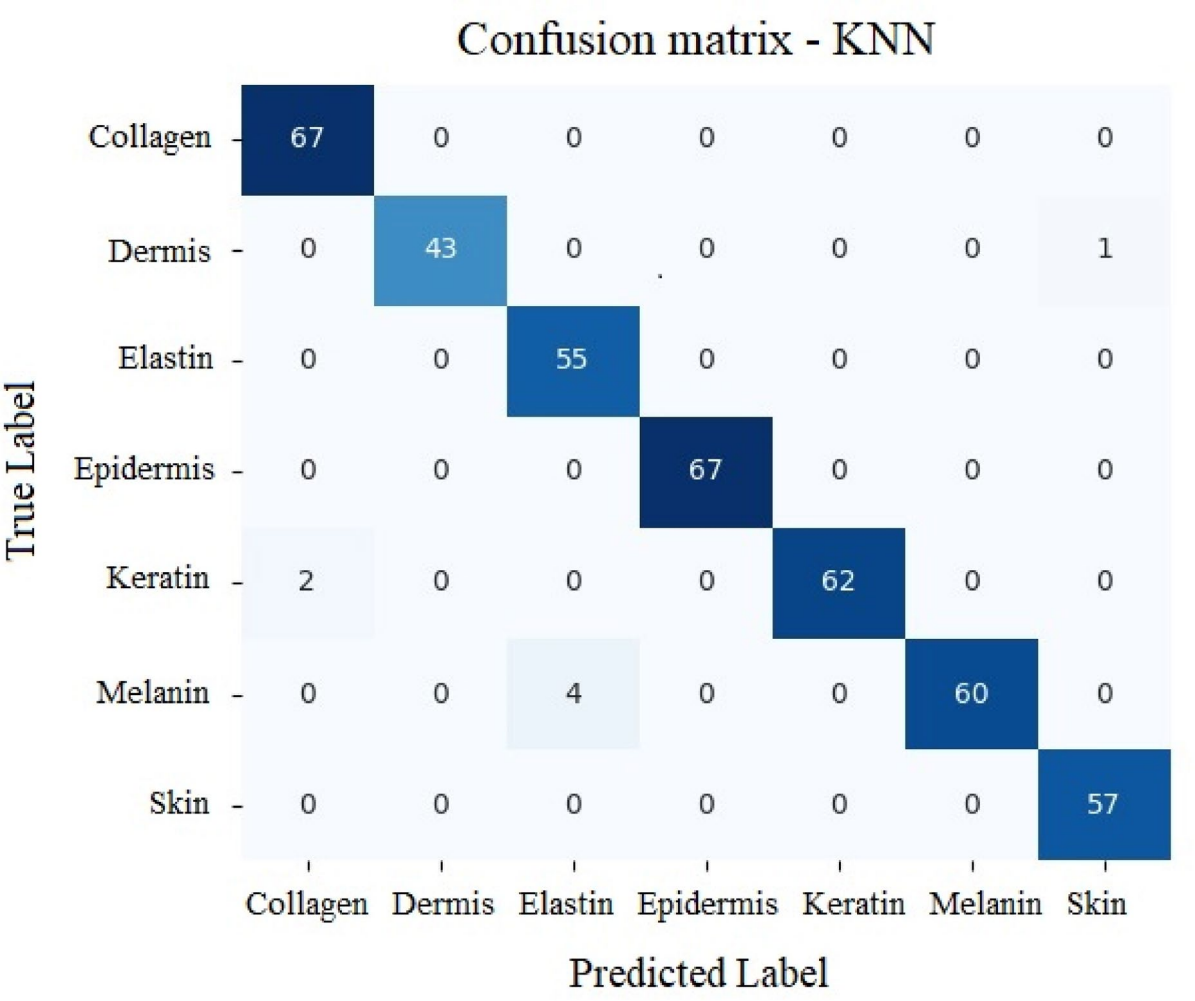
Results of KNN with respect to True vs. Predicted values.

Table 6 gives the classification report for KNN model. The accuracy provided by KNN was 98.56% to classify various analytes. Figure 23 depicts results related to functionality of KNN.

**Table 6:** Values secured by KNN for various analytes.

	Precision	Recall	F1-score	Support
Collagen	1	0.97	0.98	64
Dermis	1	0.98	0.99	57
Elastin	1	1	1	67
Epidermis	1	1	1	44
Keratin	0.95	1	0.97	55
Melanin	1	0.95	0.98	64
Skin	0.96	1	0.98	67

#### Neural network Model - Multi-Layer perceptron (MLP)

Accuracy: 98.33%

**Fig. 24:**
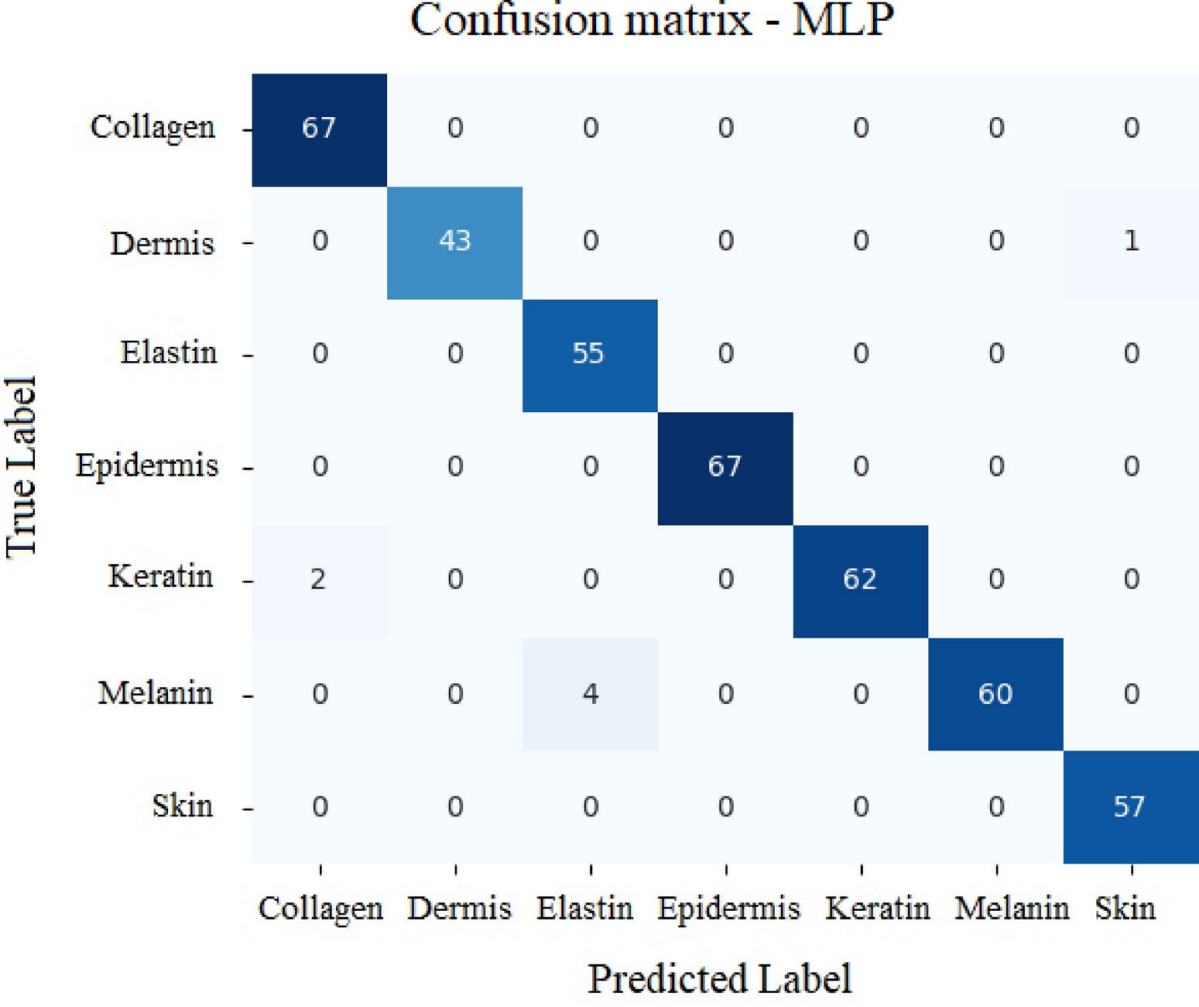
Results secured by MLP with respect to True values vs. Predicted values.

Table 7 exhibits values achieved for MLP model and Fig. 24 depicts the results for the MLP with respect to true vs. predicted values. MLP provided an accuracy of 98.33% to classify various analytes.

**Table 7:** Values secured by MLP for various analytes.

	Precision	Recall	F1-score	Support
Collagen	0.97	1	0.99	67
Dermis	1	0.98	0.99	44
Elastin	0.93	1	0.96	55
Epidermis	1	1	1	67
Keratin	1	0.97	0.98	64
Melanin	1	0.94	0.97	64
Skin	0.98	1	0.99	57

#### SVM approach

Accuracy: 96.65%

**Table 8:** Values secured by SVM model for various analytes.

	Precision	Recall	F1-score	Support
Collagen	0.97	1	0.99	67
Dermis	1	0.98	0.99	44
Elastin	0.93	1	0.96	55
Epidermis	1	1	1	67
Keratin	1	0.97	0.98	64
Melanin	1	0.94	0.97	64
Skin	0.98	1	0.99	57

**Fig. 25:**
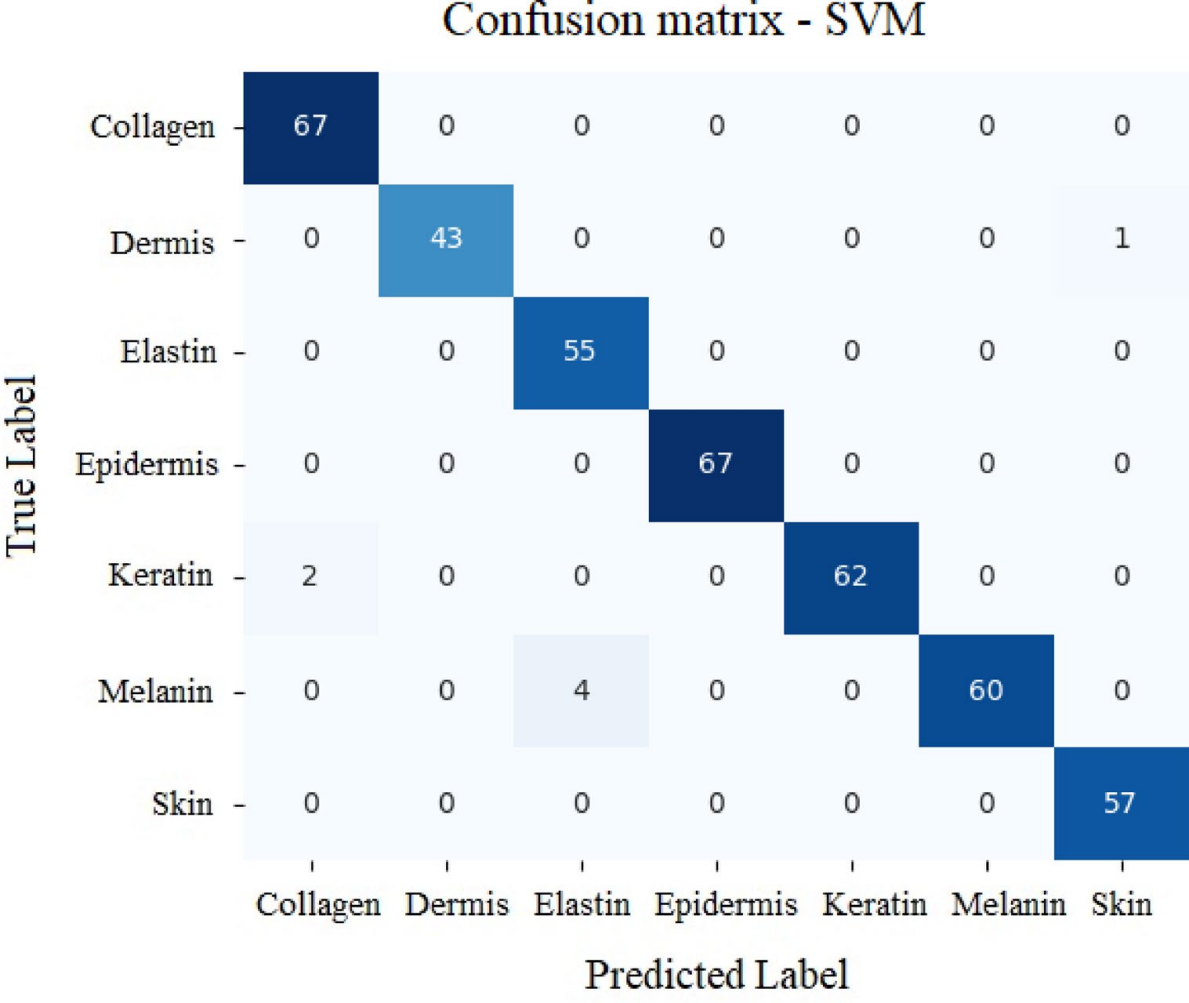
Confusion matrix for SVM model with respect to True values vs. Predicted values.

Table 8 gives the classification report for SVM model. Figure 25 shows the confusion matrix for the model. SVM approach secured 96.65% accuracy in classifying various analytes.

The main components in a confusion matrix are labels related to true and predicted values^99^. Machine learns data and predicts the class which is called as the predicted label. Whereas true label represents the class of the data obtained from the experiment or simulation.

The larger values in the diagonal elements indicate the stronger performance of the model in classifying the analytes. The values in the off diagonal cells show the instances where the classification has been incorrect.

Precision is the parameter which helps to understand how many instances were actually correct out of the predicted positive instances. The higher value of precision indicates that the false positives are less.

**Fig. 26:**
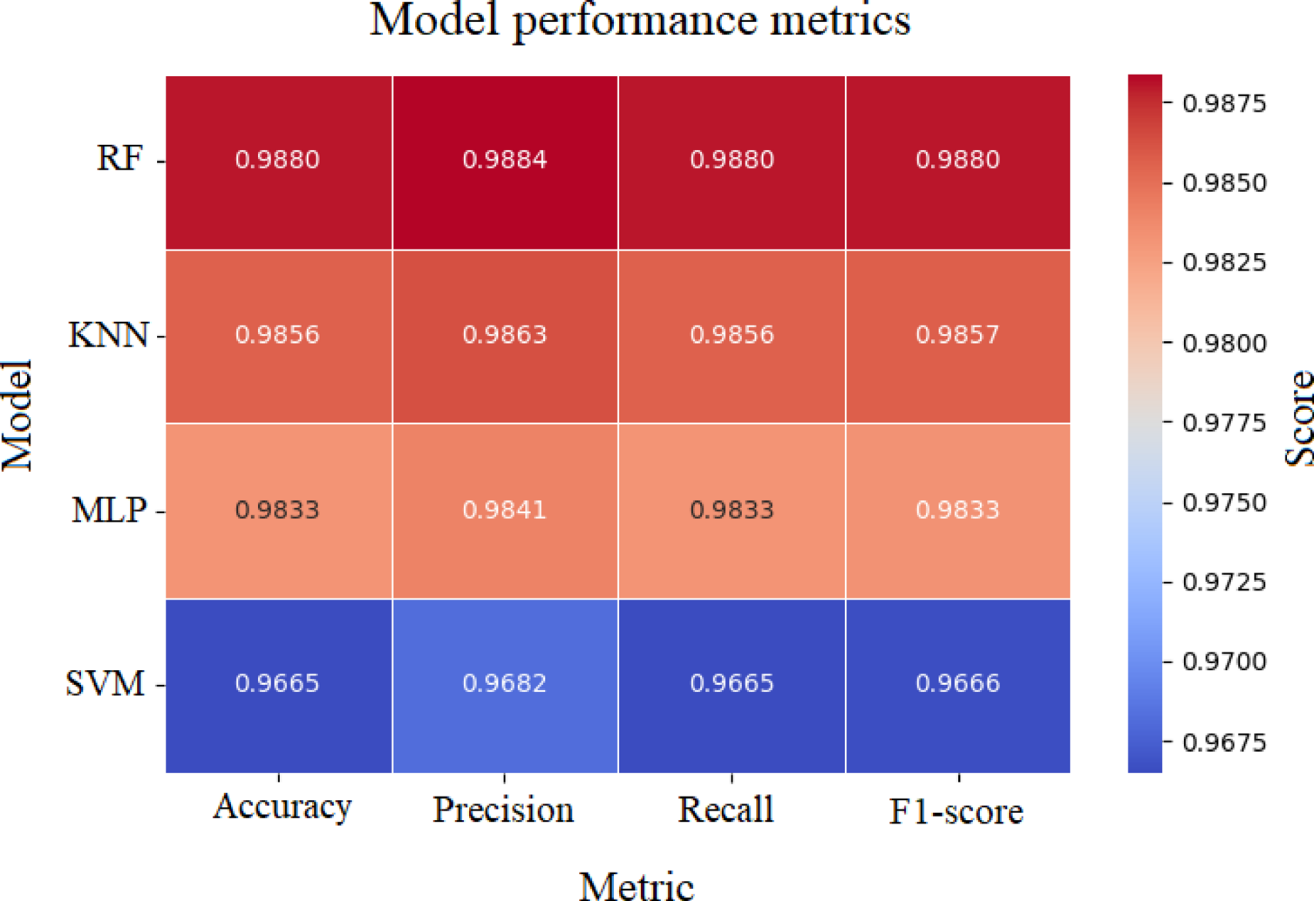
Performance comparison of various models - heat map representation.

The ability of the model to identify the actual positive instances is indicated by recall parameter. Thus, recall parameter helps to understand how many pigments are correctly identified. The F1 score is calculated using both precision and recall values^99^. The higher value of F1 score indicates that the model performed well with good precision and recall. Thus, higher F1 score means there is a better balance between the precision and recall parameter which concludes that the misclassifications are less.

The Fig. 26 shows the comparison of the parameters such as precision, recall, F1-score and accuracy. The RF classifier provided an accuracy of 98.80% each to classify the various skin analytes. The KNN and MLP algorithm provided an accuracy of 98.56% and 98.33% respectively. Support vector machine provided an accuracy of 96.65%. Thus, RF Model and KNN model can be used for the classification of the skin analytes as they provide better accuracy compared to the other two models.

### Noise modeling and cross-validation to ML datasets

Noise modeling was incorporated into ML to simulate realistic spectral fluctuations and biological variability, ensuring that the trained models are robust against measurement imperfections. Gaussian noise can be injected into the spectral data to simulate variability from placement errors, fabrication imperfections, and environmental fluctuations.

Additionally, stratified cross-validation was applied across all datasets to prevent overfitting and validate generalizability across tissue classes. The dataset included 1000 samples across 7 classes, with stratified cross-validation. Cross-validation is performed using a 5-fold Stratified K Fold approach, which divides the dataset into five equally sized subsets while preserving the original class distribution in each fold. The model is trained and evaluated five times, with each fold serving once as the test set and the remaining four as the training set. This iterative process helps mitigate the bias and variance associated with a single train-test split, offering a more reliable estimate of the model’s generalization performance. Accuracy is calculated as the average across all five folds, providing a robust summary of classifier stability.

**Fig. 27:**
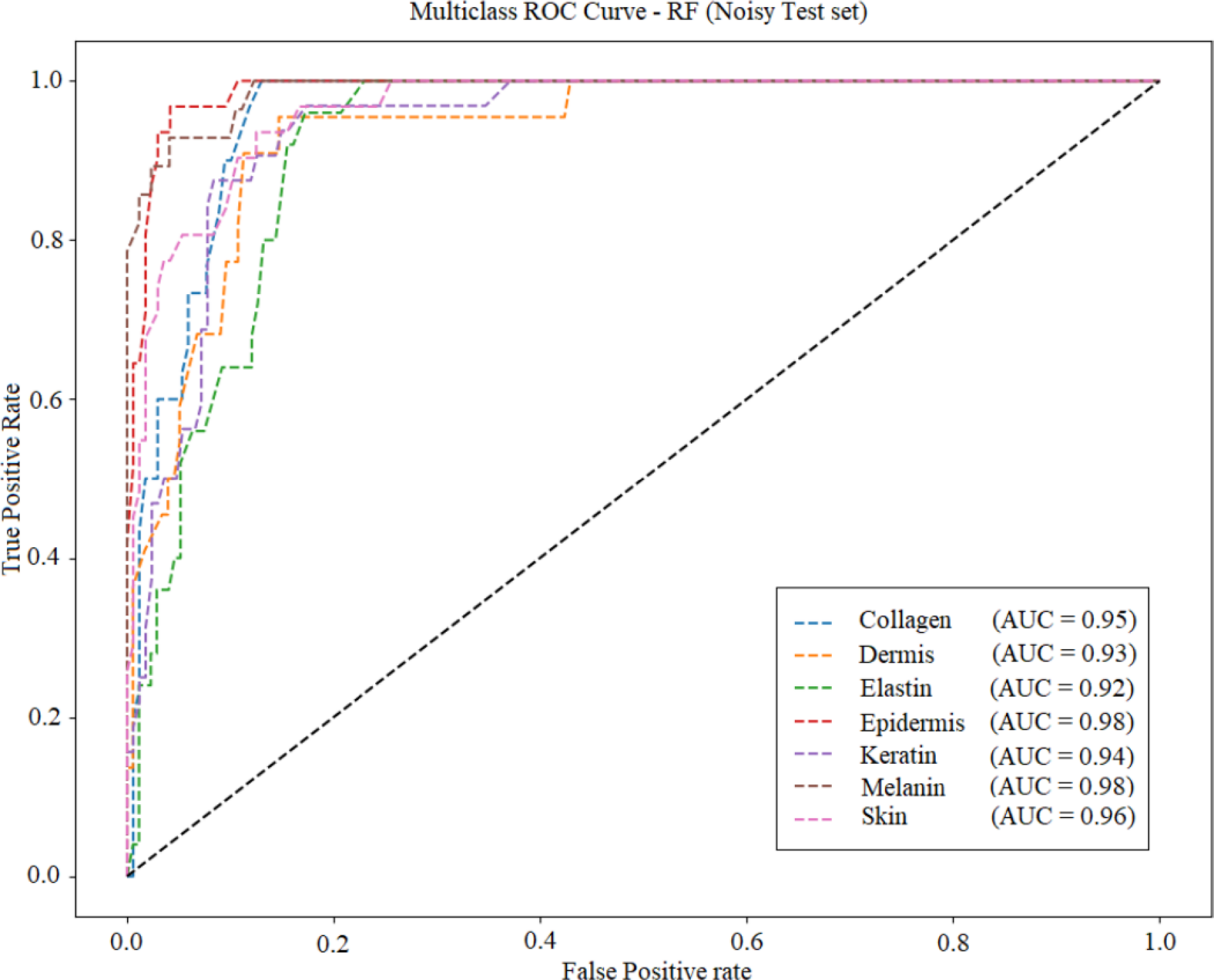
ROC curve for RF.

**Fig. 28:**
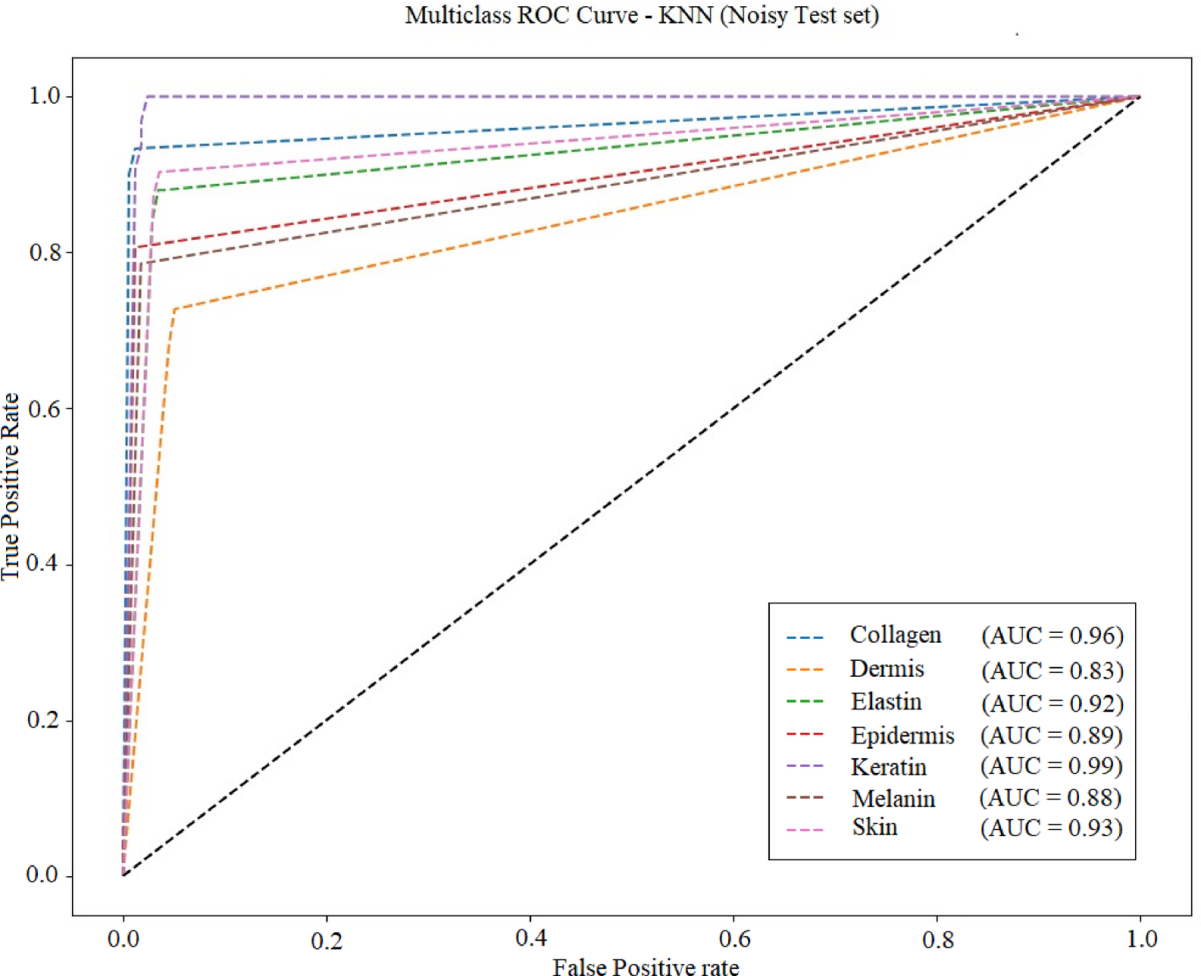
ROC curve for KNN.

**Fig. 29:**
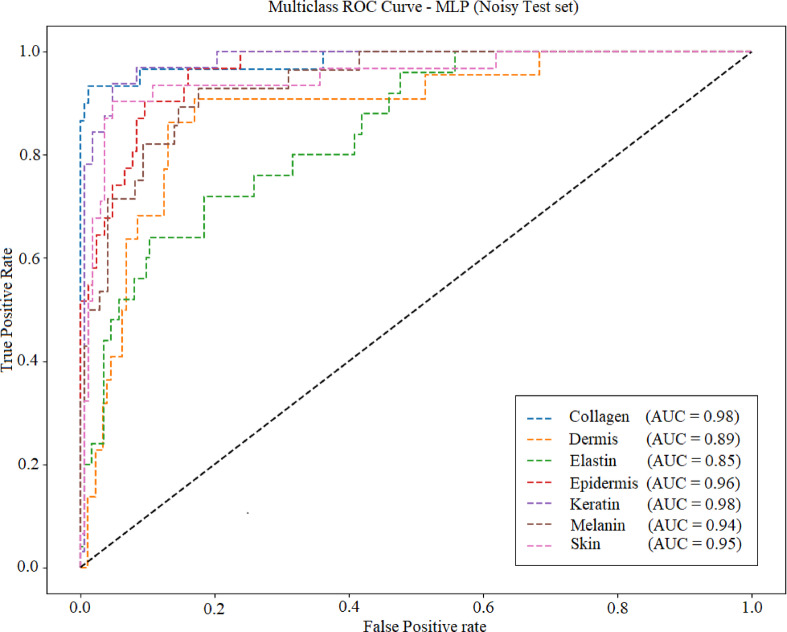
ROC curve for MLP.

**Fig. 30:**
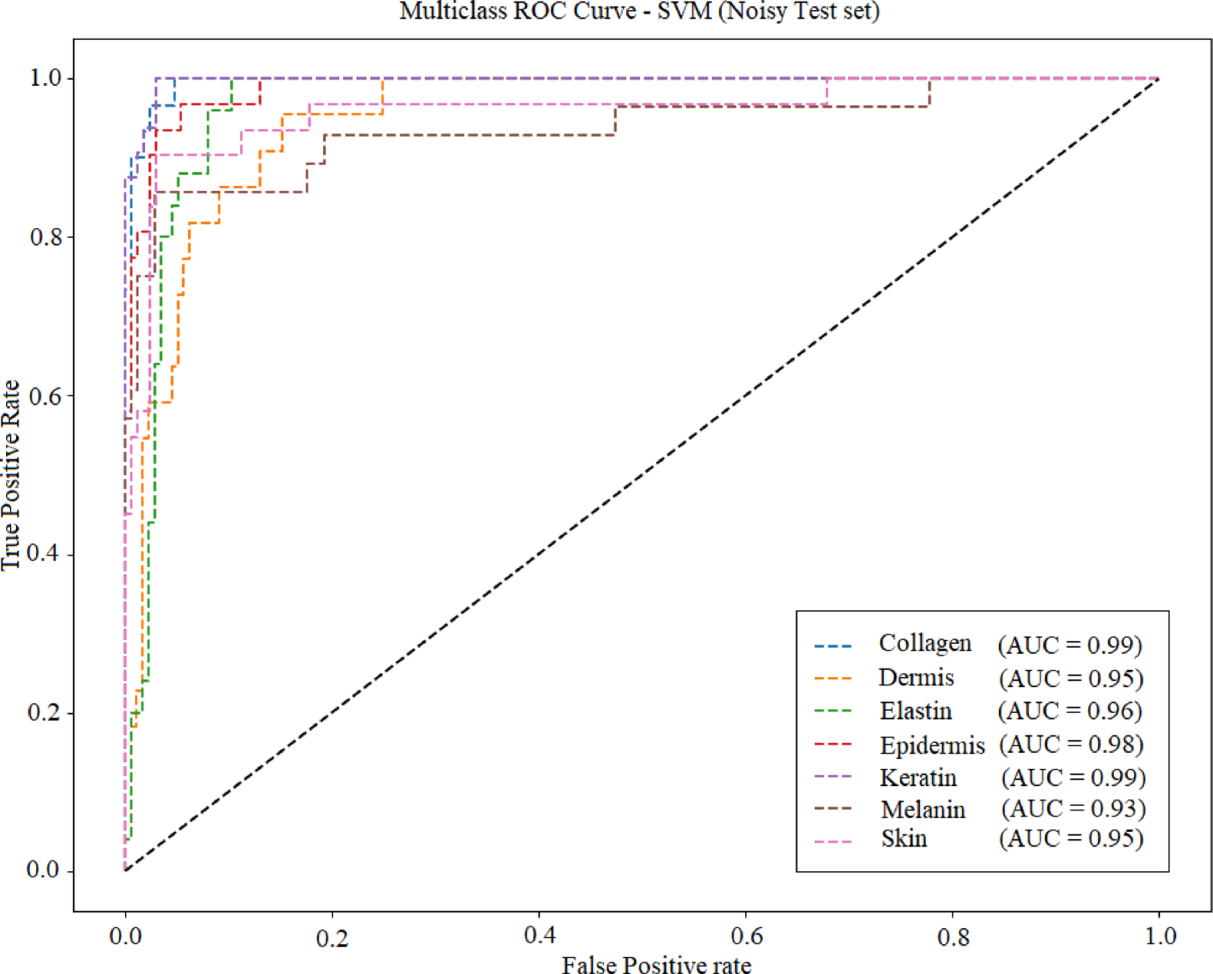
ROC curve for SVM.

Fig 27, 28, 29 and 30 displays the ROC (Receiver Operating Characteristic) curve for the four ML models under noisy conditions. AUC (area under the curve) is specified in each plot. Table 9 lists the average accuracy achieved by each model under noisy conditions. KNN model achieved average accuracy of 95.1 under the noisy conditions.

**Table 9:** Average accuracy across noise levels.

Model	Average Accuracy
KNN	95.1
SVM	94.1
RF	91.8
MLP	90.7

The advantage of ML over manual thresholding is that ML models can analyze the full spectral profile, capturing subtle variations beyond peak position or width. SVM and MLP can uncover complex patterns and correlations that manual methods may overlook, thus, nonlinear relationships can be identified. Multivariate patterns can be analyzed using ML rather than relying solely on peak positions. ML models trained on noisy or perturbed data can generalize better than fixed thresholds, especially when spectral overlap occurs as in the case of closer refractive index values. Also, ML performs classification across multiple analytes simultaneously, which becomes cumbersome with manual rules.

## Details of fabrication, experimental phase and analyte interaction with sensing region of proposed GaN PC

We acknowledge that the present study is grounded in simulation-based analysis. As part of our ongoing efforts to strengthen translational relevance, future work will focus on fabrication of GaN PC, experimental validation using tissue phantoms and spectroscopic measurement setups. Laser interference lithography, nano imprint lithography and electron beam lithography are the fabrication methods which can be applied for implementing PC structures^100^. Among the suitable substrates such as sapphire, silicon carbide (SiC), and silicon, sapphire is widely used for GaN epitaxial growth due to its lattice matching and thermal stability. The suitability of sapphire as a substrate for GaN was demonstrated in research studies published in 2016 and 2018^52,57^. GaN-on-silicon platforms are emerging as cost-effective alternatives for CMOS integration. Silane chemistry, polymer coatings, and antibody immobilization enable selective binding of biological analytes to the sensing region. Thus, functionalization can be done to immobilize skin biomarkers selectively. GaN photonic crystals can be fabricated using electron-beam lithography and reactive ion etching on GaN – on – sapphire substrates. Defect placement can be controlled via mask design.

In practical situation, to introduce skin analytes, surface contact or proximity coupling can be utilized in which the sensor is placed in direct contact with skin or exposed to skin-derived fluids. The interaction zone experiences a local RI shift, functionally equivalent to the simulated rod. In PC based sensors, microfluidic channels can be utilized to control the interaction of analytes by guiding them precisely over the sensing region^101^. Skin-derived samples can be introduced via microfluidic channels^102^. These analytes occupy or coat defect regions, altering the local dielectric environment.

In biorecognition layer functionalization, the PC surface can be functionalized with antibodies, that selectively bind skin biomarkers. Upon binding, the biomolecular layer acts as a dielectric inclusion, mimicking the rod’s optical effect. Nanoparticle or Hydrogel Embedding skin analytes can be captured in hydrogel matrices or nanoparticle carriers with controlled size (~ 0.4 μm). These carriers infiltrate the PC structure or defect zone, producing a controlled RI perturbation.

The integration of GaN 2D PC with microfluidic systems or electronic components can be complex due to limited compatibility with standard CMOS processes. These factors may contribute to production costs and scalability concerns, which must be carefully weighed against the sensor’s advantages in sensitivity, thermal stability, and potential for label-free detection. However, advances in GaN-on-silicon and GaN-on-sapphire growth techniques have significantly reduced these barriers. Despite these challenges, the integration of ML with GaN 2D PC presents a compelling opportunity for developing biosensors with enhanced diagnostic capabilities.

For testing the sensor, a tunable laser source and spectrometer can be used to measure transmission spectra. Experimental spectra can be used to retrain ML models and assess real-world performance. Exploring alternative lattice arrangements, such as hexagonal configuration, is also a direction for future work.

## Conclusion

GaN based PC biosensor is proposed for detecting skin diseases. Proposed sensor exhibited satisfactory values of DL, QF of 605, biologically relevant sensitivity of 208 nm/RIU and FOM of 59.5 RIU^− 1^. Analytes such as melanin, keratin, collagen, dermis, elastin and epidermis could be identified from the transmission spectrum. Machine learning techniques are applied in this work to classify various analytes based on the data obtained from the FDTD tool. The results showed that the incorporation of ML algorithms such as KNN, RF and MLP can be effectively used along with the PC sensor to classify the data. The QF is sufficient to resolve analytes with distinct refractive indices, as demonstrated by our ML classification results. The accuracy of the models is well suited for the detection of vitiligo and diagnosis of the skin disorders. Novelty of the work is in the use of GaN for unexplored areas such as cutis laxa using 2D PC. The sensor design can be further studied by varying the cell radius, changing the structure and also use of other wavelengths of light. The future scope of the work lies in the practical implementation of the sensor to test the sensor for real time application. The fabrication of the sensor would require expertise methods and specialized equipment with fine precision. We acknowledge retraining on experimental data will be essential for deployment in real-world settings. The current ML results serve as a proof-of-concept, demonstrating the sensor’s classification capability and guiding experimental design.

## Data Availability

The data that support the findings of this study are available upon reasonable request.
